# Dynamic heterogeneity and hidden fluidity in dense epithelial tissues

**DOI:** 10.1126/sciadv.aec3773

**Published:** 2026-04-29

**Authors:** Yuan Shen, Wang Xi, René-Marc Mège, Walter Kob, Benoit Ladoux

**Affiliations:** ^1^ Université Paris Cité, CNRS, Institut Jacques Monod, F-75013 Paris, France.; ^2^ Department of Physics, University of Montpellier and CNRS, 34095, Montpellier, France.; ^3^ Department of Physics, Friedrich-Alexander Universität Erlangen-Nürnberg, 91058 Erlangen, Germany.; ^4^ Max-Planck-Zentrum für Physik und Medizin and Max Planck Institute for the Science of Light, 91054 Erlangen, Germany.

## Abstract

Epithelial tissues maintain organ integrity while continuously remodeling during morphogenesis, repair, and disease. At high cell densities, these tissues often appear mechanically arrested in a disordered, solid-like state, raising the question of how they retain the ability to reorganize. Here, we show that, unlike thermal glasses, dense epithelial tissues do not exhibit caging behavior but instead behave as a complex fluid. Cells display subdiffusive creep together with Fickian yet non-Gaussian dynamics and compressed exponential relaxation, hallmarks of stress-driven fluidity. This fluidity arises from the tissue’s structural and mechanical organization rather than from cell division or extrusion, which only transiently enhance local dynamics. Fast-moving cells organize into collective, anisotropic clusters whose spatial heterogeneity correlates with local structural entropy and soft vibrational modes. Together, these findings reveal a hidden fluidity in densely packed epithelia that supports mechanical stability while preserving the capacity for remodeling during development, wound healing, and early tumor invasion.

## Teaser

Densely packed epithelial tissues appear mechanically arrested at tissue scale, but their cells move in subtle, coordinated ways.

## INTRODUCTION

Epithelial tissues are essential for maintaining organ integrity, guiding morphogenesis, and repairing wounds. These monolayers often undergo dynamic restructuring (*1*), yet in many physiological settings, such as mature barrier epithelia or early-stage tumors, they are packed in a dense and disordered manner and appear mechanically arrested (*2*, *3*). Such a state has often been referred to as “jammed” in previous studies (*3*–*5*), where cells are unable to rearrange freely. It has also often been discussed by analogy with glassy materials (*6*, *7*). Understanding how individual cells move within these dense tissues is crucial for elucidating how epithelial layers retain plasticity during biological processes such as morphogenesis, regeneration, and cancer progression (*8*, *9*).

At low densities, epithelial cells exhibit large-scale collective migration with continuous cell rearrangements, forming turbulent flows or flocking patterns that have been extensively characterized (*10*–*15*). As density gradually increases due to cell proliferation, cellular motion progressively slows. When crowding approaches a critical threshold, the tissue undergoes a transition from a dynamic fluid-like state to a more solid-like state, a phenomenon commonly referred to as the jamming transition (*3*–*5*). The occurrence of this transition has been reported in various biological tissues, including breast tumors (*16*, *17*), zebrafish embryos (*18*–*20*), muscle (*21*), endothelial (*22*), and epithelial tissues (*4*, *6*). These studies revealed that this transition is accompanied by complex collective cell motion (*23*), dynamical heterogeneity (*6*), and critical-like behavior (*24*). The jamming transition governs tissue patterning and compartmentalization, and its dysregulation is often linked to malignancy or developmental defects (*3*, *25*). As cell density increases further above the transition point, the tissue eventually reaches a high-density mechanically arrested regime (often termed “jammed”) in which large-scale cell rearrangements are strongly hindered and cell movements are restricted to small, local jiggling under mechanical confinement (*20*, *26*–*28*). This jiggling cell motion is often described using analogies to glass-forming materials (*6*, *26*, *28*), in which particles become trapped in “cages” formed by their neighbors (*29*). Inspired by this analogy, several theoretical models have predicted that epithelial cells in dense tissues should exhibit local confinement and slow, glassy dynamics (*24*, *30*–*37*).

However, the extent to which these models capture the true behavior of dense living tissues remains an open question. Experimentally, direct measurements of single-cell dynamics in high-density monolayers are rare (*26*, *28*), and previous observations of caging have often been inferred rather than quantified (*23*, *26*–*28*). Moreover, theoretical studies suggest that active processes, such as cell division and extrusion, can promote tissue fluidization even at high densities, thereby challenging the notion that dense tissues behave as glassy materials (*38*, *39*).

In this work, we present a comprehensive experimental investigation of the microscopic dynamics of individual cells within dense epithelial tissues. By tracking thousands of cells over long-time scales, we demonstrate that dense epithelia do not exhibit glass-like caging. Instead, they display creep motion characterized by slow, subdiffusive, anticorrelated cell movement similar to the creep behavior of amorphous materials under stress, dynamic heterogeneity, and fluid-like relaxation. We further reveal that rearrangement-prone regions show notable correlations with structural and vibrational properties, suggesting that dense tissues retain mechanical susceptibility that enables localized relaxation. These findings not only challenge prevailing assumptions about a glass state in epithelial tissues but also provide a framework for understanding how tissues balance stability with dynamic responsiveness.

## RESULTS

### Experimental setup

**Fig. 1.**
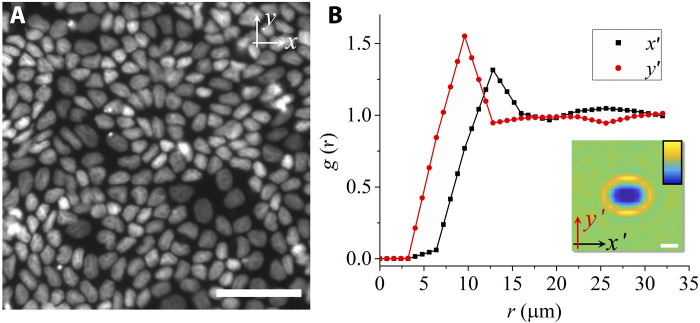
Spatial arrangement of the cells. (**A**) Microscopy image of nuclei of MDCK cells at high density. Scale bar, 50 μm. (**B**) Cross profiles of the two-dimensional pair correlation function along the *x′* axis (black squares) and *y′* axis (red circles), respectively. The two-dimensional pair correlation function (inset) of nuclei *g*(**r**) is calculated in a local coordinate frame with the long axis of the reference cell nucleus being aligned along the *x′* axis. Scale bar, 10 μm (inset). The color bar varies linearly from 0 (dark blue) to 1.5 (light yellow).

Madin-Darby canine kidney (MDCK) cells stably expressing histone1–green fluorescent protein (GFP) are cultured at high density (~9400 cells mm^−2^) on glass substrates coated with polymerized Matrigel. The samples are cultured for 2 weeks before imaging to ensure they reach a dense, mechanically arrested state. Cell nuclei are tracked using deep learning–based algorithms, achieving a tracking accuracy of ~100 nm (see fig. S1 and Materials and Methods for details). Figure 1A shows a snapshot of the cell nuclei revealing that, on large length scales, the structure is disordered, while for short distances, the packing shows a liquid-like arrangement. The inset in Fig. 1B shows the two-dimensional pair correlation function of nuclei, *g*(**r**), which measures the probability of finding another nucleus at a distance *r* from a reference nucleus, thereby characterizing the positional order of the monolayer (Materials and Methods). Here, *g*(**r**) was calculated in a local coordinate system that is aligned with the long and short axes of the nucleus, defining, respectively, the *x*′ and *y*′ axes (*29*). Figure 1B shows the corresponding one-dimensional cross profiles of the function along the *x*′ axis (black squares) and *y*′ axis (red circles), respectively. One sees that the spatial ordering of nuclei depends on the direction with respect to the orientation of the reference nucleus with a more pronounced short-range ordering along the short diameter of the nucleus (increased height of the first peak along the *y*′ axis). The location of the first peak in *g*(**r**) is about 13 μm along the *x*′ axis and 10 μm along the *y*′ axis, respectively. The oscillations of *g*(**r**) die out quickly, demonstrating a short-range positional order of the tissue (Fig. 1B). The function in the nonaligned laboratory coordinate system can be found in fig. S2 and displays the typical isotropic ring pattern found in disordered systems (*29*).

### Absence of cage effects as revealed by the mean squared displacements

**Fig. 2.**
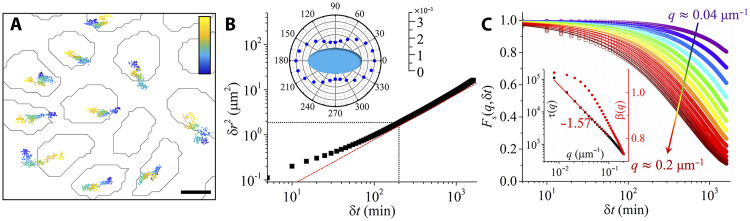
Microscopic dynamics of cells. (**A**) Trajectories of cells. Scale bar, 5 μm. The color bar scales linearly from dark blue to light yellow, which represents the time lapse from 0 to 1625 min. (**B**) TMSD of cells as a function of time delay. The dashed black lines represent the crossover from subdiffusive regime to normal diffusive regime. The red dashed line represents a power law with an exponent of 1. Inset: The diffusion coefficient of cells along different directions with respect to the long axes of the cell nuclei (unit: μm^2^ min^−1^). (**C**) Incoherent intermediate scattering function for different values of the wave vector **q**, which increases from ~0.04 to ~0.2 μm^−1^ in a step of ~0.013 μm^−1^ as indicated by the arrow. The solid lines are fits of the form $\text{exp}\left[-{\left(\frac{\delta t}{\tau \left(q\right)}\right)}^{\beta \left(q\right)}\right]$. The inset shows τ(*q*) and β(*q*) as a function of *q*. The peak in the static structure factor is around *q* ≈ 0.1 μm^−1^.

In systems exhibiting cage effects, particles remain confined within small regions for extended periods, occasionally making large jumps, often exceeding the particle size, as they hop to a new cage (*29*). However, no such behavior is observed in the nuclei motion as can be concluded from Fig. 2A, which shows representative trajectories of cell nuclei. Instead, the trajectories appear to be continuous, lacking the large, abrupt hops characteristic of cage-breaking events.

To determine quantitatively if there is a cage effect, we probe the translational mean squared displacement (TMSD), which quantifies how far, on average, individual cells move within a given time interval, thus reflecting the degree of confinement or fluidity of the tissue (Fig. 2B and Materials and Methods). For systems exhibiting cage effects, the TMSD shows a pronounced plateau at intermediate times, reflecting transient confinement that persists over a broad range of timescales (*29*, *40*, *41*). However, unlike the predictions of some vertex and Voronoi models (*24*, *32*–*37*), our data do not show such a plateau. To eliminate the possible influence of local collective cell migration related to Mermin-Wagner fluctuations, we have calculated the “cage-related TMSD” (fig. S3A and Materials and Methods), and also here, no evidence of cage effects is found. Instead, the TMSD exhibits robust subdiffusion at small δ*t* and diffusive growth at large δ*t*, confirming that the cage effect observed in thermal glassy systems is absent. Note that the overall TMSD reaches ≈20 μm^2^ (Fig. 2B), i.e., a root mean square (RMS) displacement of ≈4.5 μm. One might ask whether these displacements could arise from nuclear wobble within cells; as detailed in note S1, this interpretation is unlikely.

The anisotropic shape of cell nuclei suggests that cellular motion is not isotropic, and fig. S3C demonstrates that this is indeed the case. The inset in Fig. 2B shows the diffusion coefficient *D* as a function of the direction with respect to the long axis of cell nuclei [*D* is obtained in the diffusive regime, δ*t* = 200 to 400 min, of the TMSD according to the Einstein relation $\delta r^{2}\left(\delta t\right)=4D\delta t$]. The results show anisotropic diffusion, with a higher diffusion coefficient along the nuclear long axis.

To further investigate the dynamics in the monolayer, we analyze the incoherent intermediate scattering function $F_{s}\left(q,\delta t\right)=\langle \text{exp}\left[iq∆r_{j}\left(\delta t\right)\right]\rangle$, where $∆r_{j}\left(\delta t\right)=|r_{j}\left(\delta t\right)-r_{j}\left(0\right)|$ represents the displacement of cell *j* during the time interval δt and *q* is the wave number (*29*). This function quantifies how long a cell retains its positional memory on the spatial scale given by 1/*q*. We find (Fig. 2C) that this function decays in a simple manner and does not display the two-step relaxation that indicates the presence of cage effects (*29*, *42*). By fitting $F_{s}\left(q,\delta t\right)$ with a stretched exponential function, $\text{exp}\left[-{\left(\frac{\delta t}{\tau \left(q\right)}\right)}^{\beta \left(q\right)}\right]$, where τ(*q*) and β(*q*) are the relaxation time and stretching exponents, respectively, we find that τ(*q*) exhibits a power-law dependence on the wave vector, τ(*q*) ∼ *q*^−1.57^ (Fig. 2C, inset), deviating from the *q*^−2^ scaling found in Gaussian diffusion. This indicates that, while the system displays Fickian diffusion, i.e., the TMSD at large times grows linearly, the underlying displacement distribution is not a Gaussian. This “Fickian but non-Gaussian” dynamics has recently attracted significant attention across a variety of complex systems, including crowded intracellular environments, glassy materials, nonequilibrium systems, and fluctuations in finance and politics, etc. (*43*–*47*). Our results suggest that these unexpected dynamics, possibly arising from dynamical heterogeneity or slow but intermittent active fluctuations, govern the relaxation behavior in dense epithelial layers. At the same time, we find that at small *q*, the stretching exponent β(*q*) exceeds 1.0 (Fig. 2C, inset), indicating a compressed exponential decay of $F_{s}\left(q,\delta t\right)$. These compressed relaxations are often associated with internal stress relaxation processes, where collective rearrangements drive faster-than-diffusive dynamics (*48*). This is consistent with the presence of mechanical stresses in the dense epithelial system (*4*), where force transmission between neighboring cells can induce coordinated relaxation events. Similar compressed behaviors have been broadly observed in soft glassy materials (*49*–*51*).

In contrast to the translational dynamics of the nuclei, the rotational dynamics is subdiffusive over the entire experimental time window (fig. S3B). This persistent memory of the orientation of the cell is likely one of the reasons why their translational dynamics is Fickian but non-Gaussian for unexpectedly long times.

### Influence of cell divisions and extrusions

**Fig. 3.**
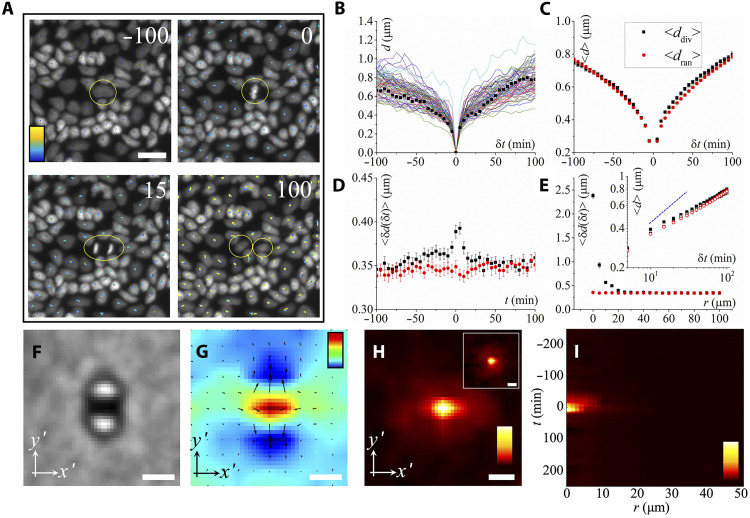
Local dynamics around divisions. (**A**) Time-lapse snapshots showing a representative cell division. Small dots indicate cell trajectories, and the color bar denotes time, scaling from 0 (dark blue) to 200 min (light yellow). The dividing cell is highlighted by a yellow circle. Scale bar, 20 μm. (**B**) Cell displacement d, of local cells surrounding a dividing cell, as a function of time. δt denotes the time relative to the division moment. Colored lines represent different division events. Black squares indicate the division shown in (A). (**C**) Mean cell displacement, <*d*>, averaged over different divisions (black squares, <*d*_div_>) as a function of time compared with random control regions (red dots, $\left\langle d_{\text{ran}}\right\rangle$). (**D**) Mean displacement step ⟨δd(δt)⟩ of local cells before and after division, averaged over divisions (black squares) and random regions (red dots). (**E**) Spatial decay of ⟨δd(δt)⟩ as a function of distance from the dividing cell. Black (red) dots denote division-centered (random) regions. Inset: log-log plot of $d_{\text{div}}\left(\delta t\right)$ and $d_{\text{ran}}\left(\delta t\right)$ from (C). Solid (open) symbols represent displacements after (before) division. The blue dashed line indicates a power-law scaling with exponent 1/2. (**F**) Averaged bright-field image of multiple divisions, aligned so that the long axis of the condensed chromosome defines the $x^{\prime }$ axis. Scale bar, 10 μm. (**G**) Mean velocity field of divisions [aligned and averaged as in (F)]. Color bar represents the divergence of velocity field that scales from −1.5 × 10^−3^ (dark blue) to 3 × 10^−3^ min^−1^ (dark red). Scale bar, 20 μm. (**H**) Two-dimensional spatial map of ⟨δd(δt)⟩ at the division moment, averaged and aligned as in (F) and (G). Inset shows the unaligned pattern. Scale bar, 10 μm. (**I**) Spatiotemporal evolution of ⟨δd(δt)⟩. Color bars scale from 0.3 (black) to 2 μm (white) in (H) and to 3 μm in (I).

Previous theoretical models have suggested that cell divisions and apoptosis might fluidize dense solid-like epithelial tissues (*38*, *39*). To test whether the absence of cage effect is due to these mechanisms, we analyze the dynamics of cells surrounding division and extrusion events (Fig. 3 for divisions and fig. S4 for extrusions). For this, we quantified the time dependence of cell displacement, *d*, averaged over cells in a square region centered on a division [extrusion] event (Fig. 3, A and B, and fig. S4, A and B). The size of the square region is 100 μm by 100 μm, which is large enough to have good statistics and also small enough to see the spatiotemporal influence of cell divisions (extrusions). We define <*d*_div_> [<*d*_ext_>] as the mean cell displacement, averaged over multiple division (extrusion) events (Materials and Methods), which quantifies the average distance traveled by the neighboring cells over time before and after divisions (extrusions). We compare it to the one of random control regions of same size, <*d*_ran_>. Figure 3C and fig. S4C demonstrate that <*d*_div_> [<*d*_ext_>] is close to <*d*_ran_> before cell division (δt<0) but becomes larger than <*d*_ran_> immediately after division [extrusion] (δt>0), indicating a local enhancement of cell motility around division (extrusion) events. A double-logarithmic plot of <*d*_div_> [<*d*_ext_>] and <*d*_ran_> reveals that both exhibit the same subdiffusive behavior (insets in Fig. 3E and fig. S4E), indicating that division (extrusion) does not change the type of cell motion in a significant manner.

This enhancement of cell mobility is also observed in the mean displacement step, 〈δd(δt=10 min)〉 (Materials and Methods), which quantifies the displacement of neighboring cells within a time span δt as a function of time before and after division (extrusion). It peaks near the division/extrusion time and decays rapidly within ~30 min (Fig. 3D and fig. S4D), confirming that the transient boost in mobility is short lived. The visualization of the averaged velocity fields also supports this transient, collective motion (Fig. 3, F and G, and fig. S4, F and G). To assess its spatial extent, we examined how 〈δd(δt)〉 varies with distance from the division (extrusion) center. We find that the boost effect is confined to ~20 μm (approximately two to three cell diameters), i.e., it is strongly localized in space (Fig. 3, E and H, and fig. S4, E and H). Both the spatial and temporal limits of this boost effect triggered by cell divisions (extrusions) can be well visualized from the spatiotemporal evolution pattern of 〈δd(δt)〉 as shown in Fig. 3I and fig. S4I.

From the above results, one thus concludes that cell divisions and extrusions can indeed locally fluidize the solid-like state of epithelial tissues. However, this effect is both spatially localized and short-lived. In addition, these events are relatively rare at high cell densities: Among ~4700 cells, we observe, on average, only one division every ~10 min and one extrusion every ~40 min. Given their low frequency and localized nature, we conclude that they are too sparse and too weak to account for the global absence of caging effects or to drive large-scale fluidization in dense epithelia, which agrees with a theoretical model reported before (*37*).

### Creep motion of individual cells

**Fig. 4.**
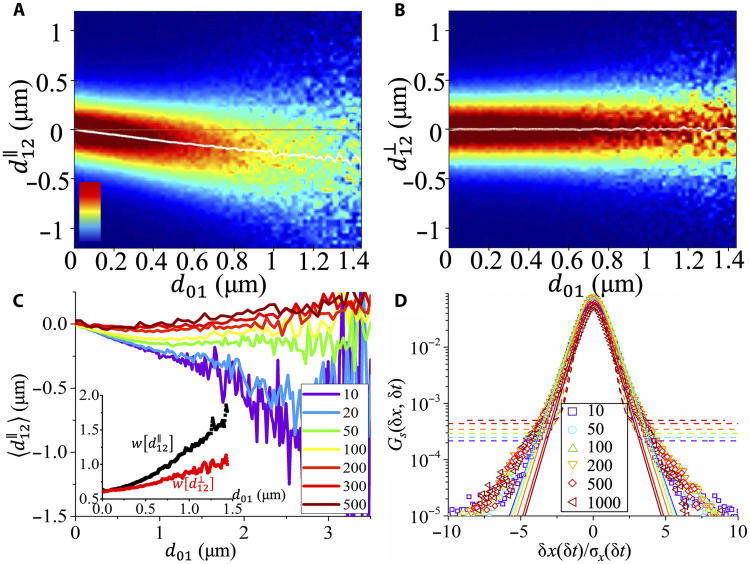
Creep motion of individual cells. (**A** and **B**) Conditional probabilities $P\left(d_{12}^{∥}|d_{01};\delta t=10\;\text{min}\right)$ and $P\left(d_{12}^{⊥}|d_{01};\delta t=10\;\text{min}\right)$ (see text for definition). The red solid lines are guides for eyes showing $d_{12}^{∥}=0$ and $d_{12}^{⊥}=0$. The white lines represent the mean values of $d_{12}^{∥}$ and $d_{12}^{⊥}$ as a function of $d_{01}$. The color bar represents the probability density which scales linearly from 0 (dark blue) to 0.05 μm^−1^ (dark red). (**C**) Mean values of $d_{12}^{∥}$ at different δt as a function of $d_{01}$. Inset: the FWHM of $P\left(d_{12}^{∥}|d_{01};\delta t=10\;\text{min}\right)$ (black) and $P\left(d_{12}^{⊥}|d_{01};\delta t=10\;\text{min}\right)$ (red) shown in (A) and (B) as a function of $d_{01}$. (**D**) Self-part of the Van Hove function for δ*x* (normalized by the square root of the mean squared displacement) at different δ*t* (expressed in minutes). The dashed and solid lines are fits with a Gaussian and a Gumbel law, respectively.

To understand the identified subdiffusive dynamics of the dense tissues, we analyze to what extent successive cell displacements are correlated in direction and magnitude. For this, we consider the function $P\left(d_{12}^{∥}|d_{01};\delta t=10\;\text{min}\right)$, which is the conditional probability of the projection of the cell displacement in the time interval [δt, 2δt] (given by $d_{12}^{∥}$) along the direction of the previous cell displacement in the time interval [0, δt] (given by $d_{01}$). (In the same way, one can define the conditional probability $P\left(d_{12}^{⊥}|d_{01};\delta t=10\;\text{min}\right)$ (*40*, *52*). Figure 4A demonstrates that the mean value of $d_{12}^{∥}$ decreases linearly from 0 to negative values as *d*_01_ increases, indicating the presence of memory effects: Cells that have moved in the direction **d**_01_ during the time interval [0, δt] are more likely to move in the opposite direction during the time interval [δt, 2δt], a transient memory effect similar to an elastic-like restoring response in which local deformations are partially reversed as stresses relax. This memory effect is also in harmony with the time dependence of the autocovariance function of cell displacement (Materials and Methods) (*53*, *54*), which shows a significant negative temporal correlation of cell motion (fig. S5). In addition, it is noted that the full width at half maximum (FWHM; Materials and Methods) of $P\left(d_{12}^{∥}|d_{01};\delta t=10\;\text{min}\right)$ increases with *d*_01_ that indicates that larger (backward) displacements are more likely for cells which have moved farther in the previous time interval (Fig. 4C, inset). On the other hand, the mean value of $d_{12}^{⊥}$ remains centered around 0 (Fig. 4B), and the FWHM of $P\left(d_{12}^{⊥}|d_{01};\delta t=10\;\text{min}\right)$ shows minimal dependence on *d*_01_ (Fig. 4C, inset), suggesting that there is no correlation between $d_{12}^{⊥}$ and **d**_01_. These results indicate that cells undergo a microscopic creep motion characterized by directionally anticorrelated steps, which may partially explain the observed subdiffusion on short timescales (Fig. 2B). In addition, it is found that as δt increases, the slope of the mean value of $d_{12}^{∥}$ gradually approaches 0 (Fig. 4C). This demonstrates that the memory effect gradually decreases with δt and vanishes when δt ~ 200 min. This timescale is consistent with the crossover of the TMSD from subdiffusive to diffusive regime (Fig. 2B), supporting the view that the subdiffusive motion is due to the memory effect. We also note that the mean value of $d_{12}^{∥}$ becomes even positive at large *d*_01_ when δt is large. This may be attributed to a local collective motion of cells that allows them to move quickly over a distance of several cell diameters. Understanding the details of this accelerated dynamics is likely important for cell migration on the mesoscopic scale, work that should be done in future studies.

More details about the microscopic relaxation dynamics of dense epithelial tissue can be obtained from the probability density function (PDF) of displacements, also known as the self-part of the Van Hove function, $G_{s}\left(\delta x,\delta t\right)$ (*29*). This function represents the probability that a cell has moved a distance δ*x* within a time interval δ*t*, thereby allowing to gain an insight into the type of motion, i.e., whether the system has a simple Gaussian dynamics or a more complex heterogeneous one. Figure 4D shows the PDF of cell displacements along the *x* axis, δ*x*, of different time steps, δ*t*. The displacements are rescaled by the RMS displacements, $\sigma_{x}\left(\delta t\right)=\sqrt{\langle \delta x{\left(\delta t\right)}^{2}\rangle }$. We observed that the rescaled curves collapse onto a master curve that cannot be well fitted with normal distribution but can at small and intermediate displacements be well described by a Gumbel law $f\left(\delta x\right)=A\left(\zeta \right)\text{exp}\left[-\frac{|\delta x|}{\zeta }\;-\;\text{exp}\left(-\frac{|\delta x|}{\zeta }\right)\right]$, where ζ is a length scale and *A*(ζ) is a normalization constant (*55*). At larger displacements, $G_{s}\left(\delta x,\delta t\right)$ displays a significant excess tail with respect to the Gumbel law, indicating an enhanced number of relaxation events on larger length scales (*56*). We find that $G_{s}\left(\delta x,\delta t\right)$ does not approach the Gaussian distribution even at large timescales, which further confirms the presence of the Fickian–non-Gaussian behavior. Together with the memory effects, our results highlight the intermittent, anisotropic nature of motion in dense epithelial tissues, where cells undergo local creep interspersed with rare, larger rearrangements. This creep motion at the single-cell scale is qualitatively distinct from the dynamics of thermal glass-forming systems and instead more closely resembles the behavior of complex fluids.

### Collective motion of cellular clusters

**Fig. 5.**
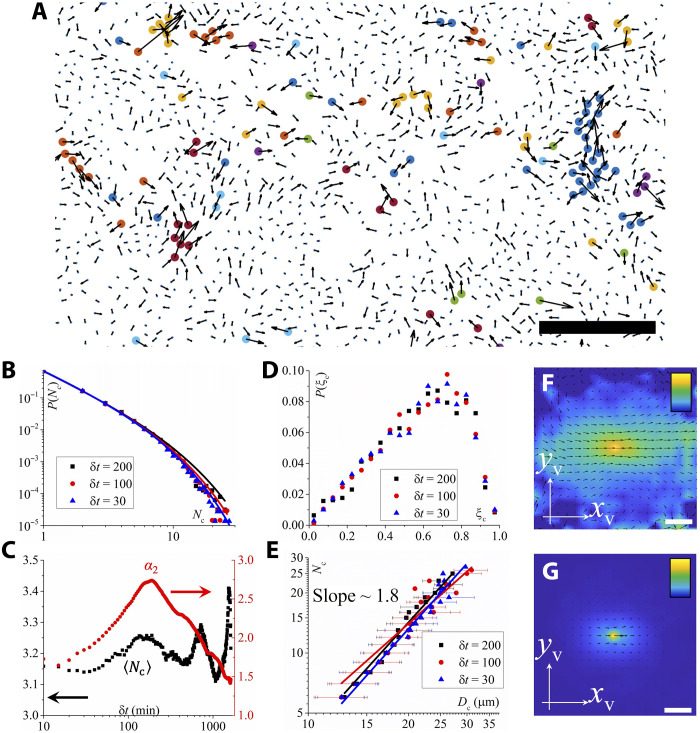
Dynamics and structure of fast cellular clusters. (**A**) Snapshot showing the dynamics of cells. The black arrows represent the displacements of cells at *t* + δt (δt = 200 min), which are magnified by three times for a better view. The small blue dots represent the positions of cells at *t*. The large circles of different colors represent different cellular clusters that move quickly (top 10%). Scale bar, 100 μm. A larger field of view can be found in fig. S8. (**B**) PDF of cluster size, *N*_c_, for different time δt. (**C**) Mean size of cellular clusters <*N*_c_> (black) and non-Gaussian parameter α_2_ (red) as a function of time step δt. (**D**) PDF of anisotropy of cellular clusters, $\xi_{c}$. (**E**) Double logarithmic plot of cluster size *N*_c_ as a function of cluster diameter *D*_c_. The solid black, red and blue lines are power law fits having exponents 1.84, 1.58, and 1.86, respectively. Two-dimensional velocity correlation functions of cells in fast clusters (**F**) and all cells (**G**) in a local coordinate system where the velocity of the reference cell is always aligned along the positive direction of the *x*_v_ axis. The color bars scale linearly from 0 (dark blue) to 1 (light yellow). Scale bars, 20 μm. The black arrows represent the mean velocity field.

In addition to the creep motion of individual cells at microscopic scales, we observe that the dynamics of the dense tissue is strongly heterogeneous as revealed by the analysis of time averaged cell displacement and diffusivity (note S2 and fig. S6). Similar dynamic heterogeneity has also been widely observed in thermal glassy systems (*42*, *57*, *58*), colloidal suspensions (*59*, *60*), and granular materials (*61*, *62*). To understand the formation of this dynamic heterogeneity and its influence on the fluidity of the dense tissues, we investigate the spatial organization of fast-moving cells. To do this, we identified the top 10% most mobile cells based on their displacement magnitude at a given time difference δt. Using a nearest neighbor criterion with a cutoff distance given by the location of the first minimum in the radial distribution function (see fig. S7 and Materials and Methods), we can group these fast cells in distinct clusters (Fig. 5A and fig. S8). Figure 5B shows the PDF of cluster sizes (the number of fast cells in a cluster), *N*_c_, at three different δt. *P*_c_(*N*_c_) first decays as a power law and then exhibits an exponential tail, which can be well described by $P_{c}\left(N_{c}\right)=A{N_{c}}^{-c}\text{exp}\left(\frac{-N_{c}}{N_{c}^{*}}\right)$, where $N_{c}^{*}$ represents the cutoff size that characterizes the transition from power law to exponential behavior ($N_{c}^{*}$ ≈ 7, 6, and 5 for δt=200,100, and 30 min, respectively). c≈1.8 is a fitting parameter, which is slightly smaller than the value of random percolation (c≈2.1) (*63*), and thus indicating the cooperative motion of cells within clusters. The prefactor A is determined from the normalization condition $\sum_{N_{c}}P_{c}\left(N_{c}\right)=1$. Similar cluster size distribution described here has also been reported in fish schools (*64*), buffalo herds (*65*), bacteria swarms (*66*), and many other animal species (*67*). Figure 5C shows the dependence of the mean cluster size (number of fast cells), <$N_{c}$>, on time, δt, and the temporal dependence of the non-Gaussian parameter $\alpha_{2}\left(\delta t\right)=\frac{\langle ∆x{\left(\delta t\right)}^{4}\rangle }{3{\langle ∆x{\left(\delta t\right)}^{2}\rangle }^{2}}-1$, where ∆x(δt) is the cell displacement along the *x* axis during the time interval δ*t*, respectively. We find that <*N*_c_> gradually increases with δt, forming a peak at δt~200 min, and then decreases with δt. (The strong oscillations at the late time stage are likely due to statistical errors caused by a few large persistent clusters.) On the other hand, the non-Gaussian parameter, α_2_, which characterizes the deviation of cell displacements from a Gaussian distribution, shows a similar temporal dependence as <*N*_c_> and forms a peak at δt∼200 min, corresponding to the end of the subdiffusive regime in the TMSD (Fig. 2B). α_2_ gets large when the cell displacement distribution has fat tails, i.e., when there is a pronounced subpopulation of “anomalously fast” cells at δt. Such a correlation between <*N*_c_> and α_2_ indicates that the motion of fast cells is cooperative. At large times, α_2_ decays to zero, but that limit seems to be reached only on timescales of 10^4^ min, i.e., a time that is unexpectedy long. Similar long-time non-Gaussianity has also been reported in various biologically relevant systems such as the motion of single amoeba cells (*46*) and colloidal cargo transport on amoeba carpets (*68*). Note that a vanishing α_2_ does not imply that the motions are Gaussian because α_2_ depends only on the second and fourth moment of the Van Hove function (*69*).

To characterize the geometry of fast-moving cell clusters, we computed their inertia tensor that quantifies how cell positions are spatially distributed within each cluster (see Materials and Methods). It allows us to compute each cluster’s anisotropy, $\xi_{c}$, and characteristic diameter, *D*_c_. $\xi_{c}=1$ for a linear structure and $\xi_{c}=0$ for a disk-like structure (*70*). These metrics allow us to quantify whether fast-moving clusters are isotropic blobs or anisotropic, string-like structures—a key question in understanding how collective rearrangements propagate in dense tissues. It should be noted that here only cellular clusters with *N*_c_ > 5 are counted. Figure 5D shows the PDF of $\xi_{c}$ for three different δt. They form a peak at $\xi_{c}\approx 0.7$, indicating an elongated shape of the clusters. In addition, the radial distribution function suggests that these clusters are elongated along their mean velocity direction (fig. S9). Furthermore, the relationship between *N*_c_ and *D*_c_ reveals that fast clusters exhibit self-similar, fractal-like behavior, $N_{c}\propto {D_{c}}^{d_{f}}$, where $d_{f}\approx 1.8$ denotes the Hausdorff dimension (Fig. 5E). This value is close to the $d_{f}$ of diffusion-limited aggregates (DLA, $d_{f}\approx 1.7$) (*71*) and the $d_{f}$ of critical percolation clusters ($d_{f}\approx 1.89$) (*72*), indicating that these cellular clusters have a ramified, porous structure that is more compact than DLA but a bit looser than critical percolation.

Last, to understand the motion of cells within clusters, we measure the velocity correlation function, $C_{v}\left(\delta r\right)=\langle v_{i}\left(0\right)v_{j}\left(\delta r\right)\rangle$, where **v** is cell velocity and δ*r* represents the distance between pairs of cells within large fast clusters ($N_{c}>5$) (Fig. 5F) and compare it with the one of the whole system (Fig. 5G). Here, both correlation functions are calculated in a local coordinate system, where the velocity of the reference cell is always aligned along the positive direction of the *x*_v_ axis. As we can see, *C*_v_ of the fast clusters is much more long-ranged compared to the one of the whole system, indicating that cells move coherently within clusters consistent with our conclusions from the correlation between <*N*_c_> and α_2_ (Fig. 5C). In addition, *C*_v_ exhibits a stronger long-range correlation along the mean cluster velocity, which is consistent with the anisotropic structure of the clusters (fig. S9).

Together, these results indicate that fast-moving cells in dense tissues are not isolated but self-organize into elongated structures that move collectively. These clusters may act as conduits for stress relaxation or as transient rearrangement pathways, enabling tissues to locally remodel without full unjamming. This collective behavior could be particularly relevant during processes such as wound healing, cancer invasion, or morphogenesis, where localized reorganization occurs within globally arrested environments (*1*–*3*).

### Microscopic origin of dynamical heterogeneity

Having established that fast-moving cells form anisotropic, dynamically collective clusters, we now investigate the origin of this dynamical heterogeneity, i.e., why do certain cells undergo large displacements and rearrangements while others remain basically static? Is this variability random, or does it reflect structurally distinct “soft spots,” akin to dislocations in crystalline solids (*73*), that are predisposed to rearrangement? While we have shown that cell divisions and extrusions can locally accelerate cellular motion, their effects are spatially localized and temporally transient. Thus, these active events alone cannot account for the widespread heterogeneity observed in the tissue.

To address this question, we quantify the degree of local rearrangement using the self-overlap parameter $Q_{i}\left(a,\delta t\right)$, which measures how far the cell *i* has moved within a time interval δ*t* compared to the length scale set by a. A value $Q_{i}\left(a,\delta t\right)=1$ indicates that the particle is still close to its initial position while a value of zero means that it has moved more than the distance a [see Materials and Methods for the definition of $Q_{i}\left(a,\delta t\right)$]. Unless otherwise noted, we use a = 1.2 μm (which is about 10% the size of a single cell) as the reference length scale and δt = 200 min as the time step to capture meaningful single-cell rearrangements at large timescales. [The temporal dependence of $Q_{i}\left(a,\delta t\right)$ of different values of a is presented in fig. S10.] We then calculate the correlation of $Q_{i}\left(a,\delta t\right)$ with various structural descriptors: local cell density, $\rho_{i}$, local nematic orientational order of nuclei, $O_{i}^{2}$, and local hexatic bond orientational order of nuclei, $O_{i}^{6}$ (Materials and Methods). Spatial maps (Materials and Methods) and Pearson correlation coefficients reveal negligible correlations between rearrangements and these standard order parameters (fig. S11), demonstrating that local density and geometric order alone do not predict dynamic activity.

**Fig. 6.**
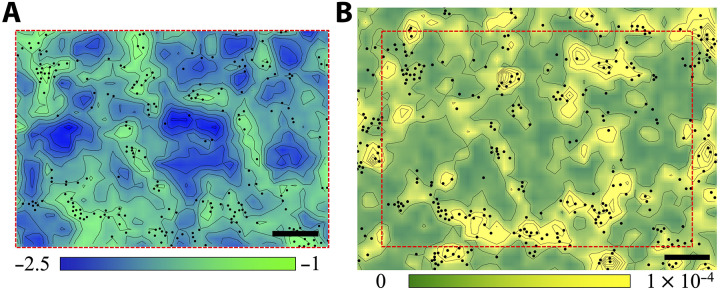
Spatial correlations between local properties and cell mobility. Contour maps of local structural entropy, *S*, (**A**) and mean magnitude of low-frequency normal modes, <*M*>, (**B**). Black dots represent the mobile cells with the 10% smallest values of self-overlap parameter $Q_{i}\left(a,\delta t\right)$. The color bars scale linearly from −2.5 to −1 for *S* and from 0 to 1 × 10^−4^ for <*M*>. Scale bars, 100 μm. The contour map of *S* is only calculated within the red dashed square region of the contour map of <*M*> to exclude the boundary effect.

In contrast, we find that local structural entropy, *S_i_* (Materials and Methods), which quantifies the degree of positional disorder in a cell’s local neighborhood (higher *S_i_* corresponds to a more disordered packing) (*74*), shows a negative correlation with $Q_{i}\left(a,\delta t\right)$ (the Pearson correlation coefficient is P≈−0.43). Cells that are persistently in high-entropy regions are more likely to undergo rearrangements (Fig. 6A), indicating that local structural disorder promotes fluidity, similar to what has been reported in thermal glassy systems (*74*, *75*). Building on this, we examined whether dynamic heterogeneity is also linked to low-frequency vibrational modes, which identify mechanically “soft” regions prone to deformation in solids (*76*, *77*). To obtain these modes, we computed the displacement covariance matrix from early-time dynamics and extracted the spatial distribution of the lowest-frequency modes (*78*, *79*). It should be noted that the measurements of the soft modes and $Q_{i}\left(a,\delta t\right)$ are done on different timescales that do not overlap (Materials and Methods for details). Cells located in regions of large vibrational amplitude are more likely to undergo rearrangements later (Fig. 6B) as demonstrated by the negative Pearson correlation coefficient (P≈−0.36) between the mean magnitude of low-frequency normal modes, <*M*>, and $Q_{i}\left(a,\delta t\right)$. This reveals a predictive link between soft vibrational modes and subsequent cellular dynamics, suggesting that the susceptibility to rearrange is encoded in the tissue’s structural and mechanical organization.

Together, these findings indicate that dynamic heterogeneity in dense epithelia arises not from local density or geometric order but from subtle variations in structural disorder and local soft vibrational modes. Considering the strong stress fluctuations in epithelial tissues reported before (*4*, *80*) and the stress driven relaxation behavior discussed above (Fig. 2C), the correlation observed here may be attributed to the large stress accumulation in regions of high structural disorder and soft vibrational modes—an intriguing hypothesis that warrants future investigation.

## DISCUSSION

In this work, we provide a comprehensive experimental characterization of the single-cell dynamics in dense epithelial tissues. Our central finding is that even at high densities, epithelial tissues are not static/rigid as thermal glassy systems. Instead, individual cells undergo subdiffusive creep motion that gradually transitions into Fickian yet non-Gaussian diffusion. This behavior suggests that even at high density, epithelial tissues remain fluid-like at the microscopic scale, continuously yielding and rearranging. Rather than being mechanically frozen, epithelia flow as complex fluids that cells explore their local environments through persistent, low-amplitude motion. These findings fundamentally revise the notion that high-density epithelia are rigid or inert, but instead align with recent simulation studies on active glass showing that persistent active forcing can fluidize dense systems without requiring cage escape or glassy relaxation dynamics found in thermal glass formers (*81*, *82*).

We further show that cell divisions and extrusions serve as local, transient sources of mechanical activity. Although they briefly accelerate the motion of neighboring cells, their influence is spatially confined and short-lived. This indicates that tissue-wide fluidity is not solely driven by active biological events but is instead an emergent feature of the tissue’s structural and mechanical organization.

Beyond the absence of caging, the system exhibits unexpected features in its displacement statistics. While cell motion is diffusive at long times, the displacement distributions remain strongly non-Gaussian, a behavior known as “Fickian but non-Gaussian” diffusion. Similar anomalous dynamics have been reported in molecular dynamics simulations of lipid membranes. These studies revealed that diffusion of lipids can emerge from collective hydrodynamic flows within the bilayer rather than from cage-escape events (*83*), whereas single-molecule studies demonstrated viscoelastic, anticorrelated subdiffusion (*84*). Furthermore, non-Gaussian displacement statistics have also been broadly observed in crowded lipid membranes (*85*, *86*). Moreover, we observe compressed exponential decays in the incoherent intermediate scattering function at low wave vectors, a signature of internal stress relaxation via collective rearrangements. These stress-driven relaxation reflects the mechanical heterogeneity and long-range force transmission within dense epithelia, highlighting the role of internal stresses as a key driver of microscopic fluidity in dense cellular assemblies.

Our identification of fast-moving, anisotropic clusters of cells provides further evidence for dynamic heterogeneity. These clusters are dynamically collective and spatially elongated along the direction of motion, resembling string-like cooperative rearrangements seen in granular and colloidal systems (*61*). We find that the occurrence of these clusters is correlated with local structural entropy and low-frequency vibrational modes—features long associated with “soft spots” in passive disordered materials (*74*, *76*). This suggests that epithelial tissues have regions predisposed to rearrangement, even in the absence of external perturbations.

From a soft-matter and biophysical perspective, our results extend the current understanding of dynamics in dense active materials. The observation that cells in dense tissues undergo subdiffusive creep and Fickian yet non-Gaussian dynamics indicates parallels to distinct active systems, such as lipid membrane (*83*–*85*), suggesting that non-Gaussian, anticorrelated subdiffusion is a general hallmark of crowded active soft-matter systems across molecular and cellular scales. Moreover, the revealed spatial correlation of dynamical heterogeneity with quantities such as the local structural entropy and soft vibrational modes extends structural-dynamical analyses from soft-matter physics to active, biological systems, providing valuable tools to gain insight into the complex structure and dynamics of biological tissues.

From a biological perspective, our findings challenge the conventional view that dense epithelial tissues are static solid-like structures, revealing a previously overlooked subtle fluidity, which ensures epithelia retain an intrinsic capacity for local reorganization and mechanical adaptability during physiological processes. During morphogenesis, for instance, epithelial sheets often undergo large-scale deformations without global unjamming (*87*); localized creep-like rearrangements and the emergence of fast cellular groups may allow tissues to respond to morphogen gradients or mechanical stresses while preserving overall cohesion. Similarly, in early carcinogenesis, subpopulations of cells may leverage this residual plasticity to initiate cell invasion and migration (*3*, *88*).

Together, our study bridges concepts from soft matter physics and epithelial biology to uncover how dense tissues sustain microscopic motion and mechanical responsiveness. By quantifying the interplay between structural disorder, internal fluctuations, and dynamic heterogeneity, we propose a previously unidentified framework for understanding the emergent mechanics responsible for the dynamics of dense epithelia. Although our findings are based on MDCK cells, the conserved structural organization of epithelial tissues suggests that similar behaviors may be present in other systems—a hypothesis warranting future investigation. These results open the door to studying tissue remodeling and soft matter-like fluidity in both physiological and pathological contexts.

## MATERIALS AND METHODS

### Cell lines and culturing conditions

MDCK cells stably expressing histone1-GFP were cultured in glass-bottom petri dishes (FluoroDish, catalog no. FD35-100) for at least 2 weeks to ensure the epithelial monolayer reached a high-density, mechanically constrained state. Cells were maintained in Dulbecco’s modified Eagle’s medium (containing glucose and pyruvate, Life Technologies) supplemented with 10% fetal bovine serum (Life Technologies) and 1% penicillin-streptomycin (Life Technologies), with medium changes every 2 days. To prepare the substrate, the glass surface of each petri dish was first plasma-cleaned for 3 min and then coated with a layer of polymerized Matrigel. To initiate polymerization, EDC [N-(3-dimethylaminopropyl)-*N*′-ethylcarbodiimide hydrochloride; E1769, Sigma-Aldrich] and NHS (*N*-hydroxysuccinimide; #130672, Sigma-Aldrich) were dissolved in cold calcium- and magnesium-free phosphate-buffered saline (PBS) and mixed with Matrigel to achieve final concentrations of 40 mM EDC and 10 mM NHS. About 50 μl of this mixed solution was smeared on the cleaned glass-bottom petri dishes and incubated at 37°C for 2 hours to allow complete polymerization. After polymerization, the coated dishes were rinsed three times with 1× PBS and incubated in 1× PBS at 37°C before cell seeding. To ensure the reproducibility of our results, all the measurements were independently replicated on two experimental samples (two biological replicates), which exhibit consistent static and dynamic behaviors (figs. S12 to S17).

### Live cell imaging and data analysis

Samples were observed through a 10× objective on a BioStation IM-Q (Nikon, Tokyo, Japan) at 37°C and 5% CO_2_ with humidification. Images were taken every 5 min for about 27 hours. More than 3800 cells were tracked over a field of view of 828 μm by 621 μm. There were overall about 4700 cells in the field of view, but ~900 of them whose trajectories were lost during the tracking over a time period of 27 hours. So, we only analyzed the rest 3800 cells whose trajectories could be tracked in all the frames. The movies were then analyzed through Cellpose2.0 (*89*), ImageJ, and MATLAB. Nuclei were first segmented by Cellpose, which were then tracked using the Trackmate plugin of ImageJ (*90*). The tracking results (including the spatial coordinates of the center of mass of cell nuclei and the long-axis orientation of cell nuclei) were then analyzed through MATLAB.

To characterize the tracking error produced during the experiment, we compare the cell displacements of dense tissues with a fixed sample where cells are dead and thus do not move as shown in fig. S1. It is found that the tracking error is basically independent of time and is around 0.1 μm, which is less than the half the cell displacements at the smallest δ*t*. At the same time, cell nuclei can change their shapes over time. To characterize its influence on the dynamics, we calculate the mean values of the changes of the long and short axes of cell nuclei as a function of time, $\delta d_{\alpha }\left(\delta t\right)=\langle d_{\alpha }^{i}\left(t+\delta t\right)-d_{\alpha }^{i}\left(t\right)\rangle$, where $d_{\alpha }^{i}\left(t\right)$ represents the length of the long (α=l) or short (α=s) axis of nucleus *i* at time *t* and 〈…〉 represents averaging over different cell nuclei at different times. It is found that both of them are much smaller compared to the cell displacements and become negligible at large δ*t*. So, we conclude that the tracking error of our experiments is around half of the length of cell displacements at the smallest δ*t* and becomes negligible as δ*t* increases.

#### Pair correlation function

The pair correlation function is calculated as $g\left(r\right)=\frac{1}{\rho N}\langle \sum_{i\neq j}\delta \left[r-(r_{i}-r_{j})\right]\rangle$, where ρ is the cell density of the tissue, *N* represents the total cell number, **r***_i_* represents the position of cell *i*, and 〈…〉 represents an average over configurations at different times.

#### Translational mean squared displacement

The TMSD is calculated as $\langle {\delta r}^{2}\left(\delta t\right)\rangle =\langle {\left[r_{i}\left(t+\delta t\right)-r_{i}\left(t\right)\right]}^{2}\rangle$, where $r_{i}\left(t\right)$ is the position of cell *i* at time *t*. The cage-related TMSD is defined as, $\langle {\delta r}_{c}^{2}\left(\delta t\right)\rangle =\langle {\left\{\left[r_{i}\left(t+\delta t\right)-r_{i}\left(t\right)\right]-\frac{1}{n_{i}\left(t\right)}\sum_{j}^{n_{i}\left(t\right)}\left[r_{j}\left(t+\delta t\right)-r_{j}\left(t\right)\right]\right\}}^{2}\rangle$, where $n_{i}\left(t\right)$ is the number of nearest neighbors of cell *i* at time *t*, the nearest neighbors are defined as cells whose distances between themselves and the reference cells is smaller than the distance of the first peak of the radial distribution function of the system.

#### Cell dynamics of division/extrusion events

The mean displacement of cells over different division [extrusion] events is defined as $d_{\beta }\left(\delta t\right)=\langle |r_{\beta }^{i}\left(\delta t\right)-r_{\beta }^{i}\left(0\right)|\rangle$, where $r_{\beta }^{i}\left(\delta t\right)$ represents the spatial coordinate of cell *i* in the division (β=div)/extrusion (β=ext) region at time δ*t* away from division [extrusion] event, 〈…〉 represents average over different local cells around division [extrusion] events, and δ*t* represents the time delay before and after the division [extrusion] moment. The mean displacement step is defined as $\langle \delta d\left(\delta t\right)\rangle =\frac{1}{n}\sum_{j}^{n}\frac{1}{n_{j}}\sum_{i}^{n_{j}}|r_{i}\left(t+\delta t\right)-r_{i}\left(t\right)|$, where **r***_i_*(*t*) represents the position of cell *i* around division [extrusion] event *j* at time *t*, δ*t* = 10 min represents time interval, *t* represents the time moment away from the division moment, and *n_j_* and *n* represent the number of cells around division event *j* and the number of division events, respectively.

#### Autocovariance function of cell displacement

The autocovariance function of cell displacement is defined as $C_{\tau }\left(\delta t\right)={\langle {\hat{u}}_{i}^{\tau }\left(\delta t\right)\cdot {\hat{u}}_{i}^{\tau }\left(0\right)\rangle }_{i,t}$, where ${\hat{u}}_{i}^{\tau }\left(\delta t\right)=u_{i}^{\tau }\left(\delta t\right)/|u_{i}^{\tau }\left(\delta t\right)|$, $u_{i}^{\tau }\left(\delta t\right)=r_{i}\left(\delta t+\tau \right)-r_{i}\left(\delta t\right)$, δ*t* represents time delay, τ represents the bin time, **r***_i_* represents the position of cell *i*, and ${\langle \ldots \rangle }_{i,t}$ represents an average over cells and time.

#### Memory function

To calculate the FWHM of $P\left(d_{12}^{∥}|d_{01};\tau =10\right)$ [$P\left(d_{12}^{⊥}|d_{01};\tau =10\right)$], we first calculate the second moment $m_{2}=\sum_{j}w_{j}{\left(x_{j}-\bar{x}\right)}^{2}/\sum_{j}w_{j}$, where $\bar{x}=\sum_{j}w_{j}x_{j}/\sum_{j}w_{j}$, *x_j_* represents the values of $d_{12}^{∥}$ ($d_{12}^{⊥}$), and *w_j_* represents the corresponding probability weight of *x_j_*. The FWHM is then defined as $w\left[d_{12}^{∥,⊥}\right]=2\sqrt{2\text{ln}\left(2\right)\times m_{2}}$.

#### Cellular clusters

To define the clusters of fast cells, we first calculate the cell displacements at each time as $d_{i}\left(t,\delta t\right)=|r_{i}\left(t+\delta t\right)-r_{i}\left(t\right)|$ and define the top 10% of cells having the largest $d_{i}\left(t,\delta t\right)$ as fast cells. We then define a threshold distance r* = 15 μm, which is the distance of the first valley of the radial distribution function of the epithelial system (fig. S7). Two fast cells are considered to be neighbors if their intercellular distance is smaller than r* and a fast cell belongs to a fast cellular cluster if it is the neighbor of any other fast cells belonging to the cluster.

The inertia tensor is defined according to (*70*, *91*), $M_{\text{ab}}=\sum_{i=1}^{N_{c}}\left(r_{a}^{i}-\bar{r_{a}}\right)\left(r_{b}^{i}-\bar{r_{b}}\right)$, where *a*, *b* represents *x*, *y*; $r^{i}=\left(r_{x}^{i},r_{y}^{i}\right)$ are the spatial coordinate of the *i*th cell; and $\bar{r}=\frac{1}{N_{c}}\sum_{i=1}^{N_{c}}r^{i}$ is the center of mass of the cluster. *M*_ab_ allows to define a characteristic diameter, $D_{c}=2\sqrt{\text{Tr}\left(M\right)/N_{c}}$, and anisotropy of the structure, $\xi_{c}=\left|\frac{\left(M_{\mathrm{xx}}-M_{\mathrm{yy}}\right)}{\left(M_{\mathrm{yy}}+M_{\mathrm{xx}}\right)}\right|$.

#### Non-Gaussian parameter

The non-Gaussian parameter that quantifies the deviations from a Gaussian distribution is defined as $\alpha_{2}\left(\delta t\right)=\frac{\langle ∆x{\left(\delta t\right)}^{4}\rangle }{3{\langle ∆x{\left(\delta t\right)}^{2}\rangle }^{2}}-1$, where ∆x(δt) is the cell displacement along the *x* axis during the time interval δ*t*.

#### Self-overlap parameter

The self-overlap parameter is defined as $Q_{i}\left(a,\delta t\right)={\langle \text{exp}\left(-\frac{{|r_{i}\left(t+\delta t\right)-r_{i}\left(t\right)|}^{2}}{2a^{2}}\right)\rangle }_{t}$, where *i* represents the specific cell and *a* is the reference length scale. $Q_{i}\left(a,\delta t\right)$ decays from 1 to 0 as cells gradually move away from their initial reference positions. It should be noted that here $Q_{i}\left(a,\delta t\right)$ is averaged over time from *t* = 105 min to *t* = 1625 min to reduce the noise. The reason that we can do this time average is because the cells are not moving that much within this time span, i.e., they do not change their nature (say from fast to slow) as shown in Fig. 2A. So, the time average improves the statistics but does not wipe out the information of interest.

#### Nematic order parameter

The local nematic order parameter (*92*) is defined as $O_{i}^{2}=\sqrt{{\langle Q_{\mathrm{xx}}\rangle }^{2}+{\langle Q_{\mathrm{xy}}\rangle }^{2}}$, where $Q_{\mathrm{xx}}=\text{cos}\left(2\theta \right)$ and $Q_{\mathrm{xy}}=\text{sin}\left(2\theta \right)$ are components of nematic tensor **Q**, θ is the angle between the long axis of cell nuclei and a fixed axis, and 〈…〉 represents the average of the tensor **Q** over the reference cell *i* and its Voronoi neighbors.

#### Hexatic order parameter

The hexatic bond orientational order parameter is defined according to ref. (*93*), $O_{i}^{6}=\left|\frac{1}{n_{i}}\sum_{k=1}^{n_{i}}e^{j6\gamma_{\mathrm{ik}}}\right|$, where *n_i_* is the number of Voronoi neighbors of cell *i* and γ*_ik_* represents the angle between a reference axis and the bond vector connecting cell *i* and its neighbor *k*.

#### Local structural entropy

The local structural entropy is defined according to (*74*), $S_{i}=-\frac{\rho }{2}\int \mathrm{dr}2\pi r\left\{g_{i}\left(r\right)\text{ln}g_{i}\left(r\right)-\left[g_{i}\left(r\right)-1\right]\right\}$, where *g_i_*(*r*) is the radial distribution function of cell *i*. Similar to $Q_{i}\left(a,\delta t\right)$, the contour maps of $\rho_{i}$, $O_{i}^{2}$, $O_{i}^{6}$, and *S_i_* are averaged over the time period from *t* = 105 min to *t* = 1625 min to improve statistics.

#### Vibrational modes

The method used for the calculation of the vibrational modes can be found in (*78*, *94*). In brief, to find the low-frequency normal modes, we construct the covariance matrix, $D_{\mathrm{kl}}={\langle u_{k}u_{l}\rangle }_{t}$, where $u_{k}=x_{k}-{\langle x_{k}\rangle }_{t},y_{k}-{\langle y_{k}\rangle }_{t}$, and k,l=1,2,…,2N (*N* cells) run over all cells and their Cartesian components in two dimensions, based on the cell displacements in the first 100 min over which there are few cell rearrangements. We then obtain the eigenvalues λ*_k_* and their corresponding eigenmodes **M***_k_*. We average the magnitude of the 10 eigenmodes that have the 10 largest eigenvalues (lowest frequencies $\omega_{i}=\sqrt{\frac{1}{\lambda_{i}}}$) to get the contour map of mean magnitude of low-frequency normal modes, <*M_k_*>, and compare it with $Q_{i}\left(a,\delta t\right)$. It should be noted that here the self-overlap parameter, $Q_{i}\left(a,\delta t\right)$, is calculated on the basis of the time window after the first 100 min, i.e., $T_{2}=\left[105,1625\right]$ min, so there is no overlap between the calculations of the covariance matrix **D** and the self-overlap parameter $Q_{i}\left(a,\delta t\right)$.

#### Colormaps

To plot the colormap of $\rho_{i}$, $O_{i}^{2}$, $O_{i}^{6}$, *S_i_*, and <*M*>, we applied a coarse-graining method. Basically, we first divide the whole field of view of our data into small windows (40 μm by 40 μm), with an overlap of 20 μm between the windows. Then, in each small window, we average the values of the specific parameter of the cells within that window. Last, we interpolate those mean values and get the colormaps.

## Supplementary Materials

### This PDF file includes:

Notes S1 and S2 Figs. S1 to S17 References
