# Supplementary material for: Dynamic heterogeneity and hidden fluidity in dense epithelial tissues
Source: Sci Adv. 2026 Apr 29;12(18):eaec3773. doi: 10.1126/sciadv.aec3773 (PMC13127560; doi:10.1126/sciadv.aec3773)
Supplement: Supplementary file 1 — Notes S1 and S2 Figs. S1 to S17 References [file sciadv.aec3773_sm.pdf]

Science Advances
AAAS

Supplementary Materials for

Dynamic heterogeneity and hidden fluidity in dense epithelial tissues

Yuan Shen *et al.*

Corresponding author: Yuan Shen, yuan.shen@ijm.fr; Walter Kob, walter.kob@umontpellier.fr;
Benoit Ladoux, benoit.ladoux@fau.de

Sci. Adv. **12**, eaec3773 (2026)
DOI: 10.1126/sciadv.aec3773

This PDF file includes:

Notes S1 and S2
Figs. S1 to S17
References

Supplementary Notes

Supplementary Note 1: Excluding intra-cell nuclear wobble as the origin of MSD.

1. **Geometric upper bound.** At our confluence (≈ 9,400 cells mm^{-2}), the mean lateral cell footprint is ≈ 106 µm^2 (equivalent circular diameter ≈ 11.6 µm). With nuclear diameters of order 8–10 µm, simple geometry bounds any intra-cell nucleus-centroid shift to $\Delta r_{\text{intra}}^{\max} \leq (d_{\text{cell}} - d_{\text{nucleus}})/2 \approx$ 1-2 µm before contacting the cortex. This 1–2 µm value is a conservative geometric ceiling; cytoskeletal tethering and cytoplasmic viscoelasticity make typical intra-cell nuclear fluctuations substantially smaller, whereas we measure ~4.5 µm RMS—well beyond intra-cell wobble and thus indicative of tissue-level motion.
2. **Tracking/shape controls.** We quantified the tracking error on fixed samples as ~0.1 µm, time-independent, and also showed that the fluctuations of nuclear long/short axes are much smaller than the measured displacements. A 4–4.5 µm displacement is 40 times larger than the tracking error and far above nuclei shape-change artifacts.
3. **Independent signatures of tissue-level dynamics.** Our analysis disfavors the wobble hypothesis: (i) the TMSD shows no long-time plateau expected for a nucleus caged in a cell (Ornstein–Uhlenbeck) (Fig. 2b), (ii) the incoherent intermediate scattering function exhibits compressed relaxation at small *q* (Fig. 2c), (iii) divisions/extrusions induce transient flow over ~20 µm (Fig. 3), (iv) short-time steps display directional anti-correlations (creep-like memory; Fig. 4a), (v) velocity–velocity correlations extend to ~20 µm (Fig. 5f-g), and (vi) rearrangements correlate with structural entropy and soft vibrational modes (Fig. 6). Together, these are signatures of mechanically coupled, tissue-level dynamics rather than independent nuclear jiggle.

Supplementary Note 2: Analysis of time averaged cell displacement and diffusivity.

To characterize the dynamic heterogeneity of the jammed epithelial tissue, we measured the time averaged displacement of individual cells (*85*,*95*) defined as

$$\overline{\Delta_i^2(\delta t, T)} = \frac{1}{T-\delta t}\int_0^{T-\delta t} [\mathbf{r}_i(t+\delta t) - \mathbf{r}_i(t)]^2\, dt,$$

where $\delta t = 50$ min and $\mathbf{r}_i$ represents the position of cell *i*. The *T*-dependence of this quantity is presented in Fig. S6a. We recognize that individual time-averaged MSD curves fluctuate randomly, whereas their mean (thick black line) is independent of *T*, as expected for a system in its steady state. We note that this time-dependent mobility varies from cell to cell by orders of magnitude, confirming the strong dynamical heterogeneity of the tissue. Within the time scales accessible to our experiment, these fluctuations do not decay to zero, demonstrating that the dynamical heterogeneities are very long lived. The spread of this distribution of $\overline{\Delta_i^2(\delta t, T)}$ can be quantified by the "ergodicity breaking parameter" (*EB*) (*85*,*95*) defined as

$$EB(T) = \left[\langle\left(\overline{\Delta^2(T)}\right)^2\rangle - \langle\overline{\Delta^2(T)}\rangle^2\right]/\langle\overline{\Delta^2(T)}\rangle^2.$$

We have measured *EB*(*T*) in two different randomly selected regions (130 µm ×130 µm, including 206 and 189 cells, respectively) and present the resulting time dependence in Fig. S6b. One sees that *EB*(*T*) depends strongly on the region in that for region #1 (blue stars), one finds that *EB*(*T*) is basically constant up to 90 min and then starts to decay like T^{-1}. The subsequent 1/*T* dependence is the one expected from the central limit theorem. In contrast to this behavior we find for region #2 (red crosses) an *EB*(*T*) that is significantly smaller (by a factor of 10) and which decays quickly to a value of *O*(1) before it fluctuates around this value. Also, the system averaged *EB*(*T*) (black circles) is constant up to around 90 min before it shows the expected 1/*T* decay. Note that these curves are subject to a relatively large error and hence we refrain from extracting exponents from the apparent power-laws of the decays.

From the local slope of the time-dependent displacement of the individual cells, one can obtain a time-dependent effective diffusion constant according to Refs. (*85*,*95*):

$$D(\delta t, T') = \frac{\overline{\delta r_i^2(\delta t, T')}}{4\delta t}, \text{ where } \overline{\delta r_i^2(\delta t, T')} = \frac{1}{\delta t}\int_{T'}^{T'+\delta t} [\mathbf{r}_i(t+\delta t) - \mathbf{r}_i(t)]^2\, dt,\ \delta t = 50 \text{ min}.$$

In Fig. S6c we show the time dependence of $D(\delta t, T')$ for different cells and one sees that this quantity depends significantly on the cell considered, indicating the strong dynamical heterogeneity of the tissue. However, its time-dependence is relatively weak, in agreement with the view that on the time scale of our experiments the dynamical heterogeneities do not evolve strongly. The distribution of $D(\delta t, T')$ is shown in Fig. S6d and one

sees that it has a single maximum with a tail at large $D(\delta t, T')$ that is compatible with a power-law with an exponent around -3. (We have checked that this exponent does not depend significantly on the time lag δt used for the coarse-graining.) At present we do not have any explanation for this value and understanding it remains thus work for the future. Also, the absence of a double peak structure in the distribution function, a feature found in the corresponding quantity in systems of lipid bilayers (*1*), indicates that our cell tissue does not have two distinct states that differ in their dynamics, but instead a broad distribution of temporal mobility.

Supplementary Figures

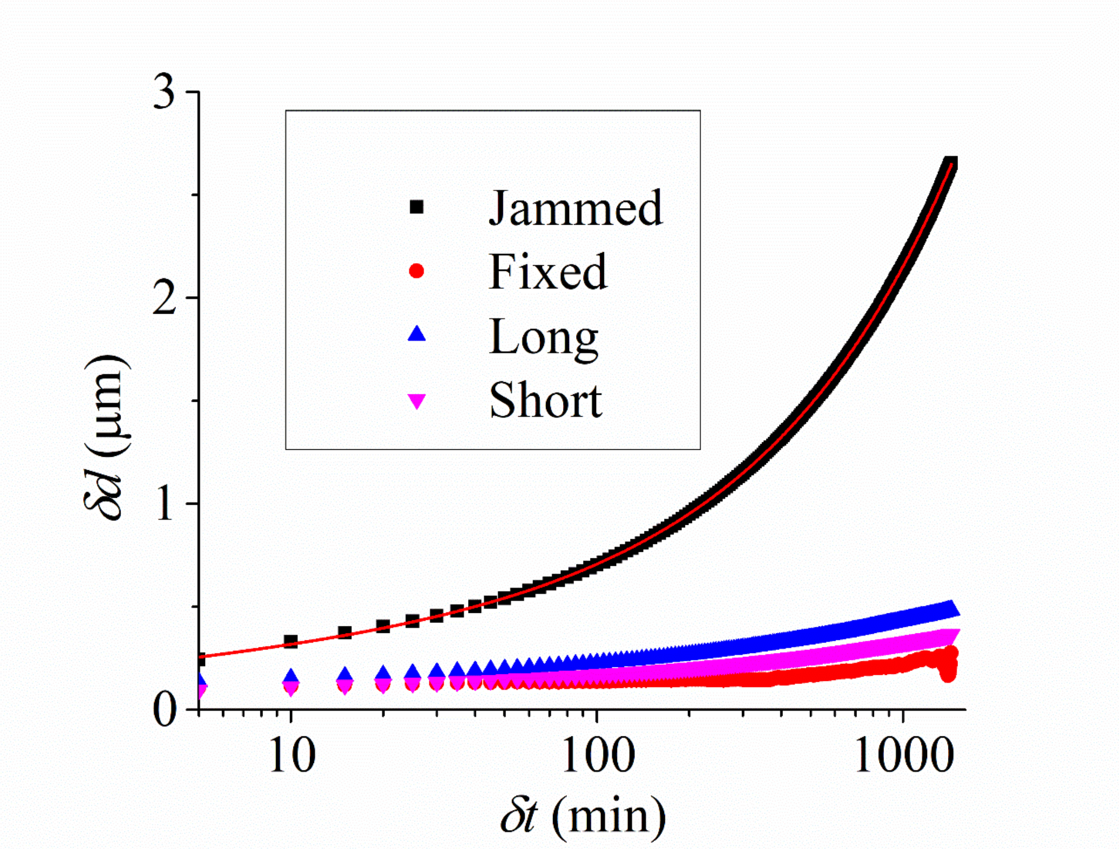

Fig. S1 Cell displacement as a function of time delay. The black squares represent the displacements of cells in the jammed epithelial sample. The data is fitted by a nonlinear function (red solid line) $\delta d = a \times \delta t^b + c \times \ln(f \times \delta t)$, where a = 0.014, b = 0.693, c = 0.053 and f = 11.689. It can be speculated that this functional form is related to an anomalous transport of the particles (power-law) enhanced by rare escape events that involve the crossing of a barrier (logarithmic dependence). The red circles represent the displacements of fixed (dead) cells. Blue and pink triangles represent the changes of the lengths of the long and short axes of cell nuclei as a function of time delay. One recognizes that the displacement of cells in the jammed tissue is significantly larger than the one of the potential error sources.

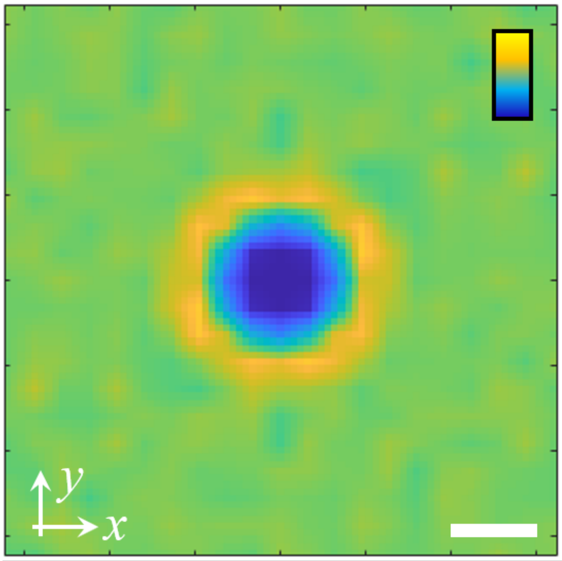

Fig. S2 Pair correlation function of nuclei in the non-aligned lab coordinate frame. The scale bar is 10 µm. The color bar scales linearly from 0 (dark blue) to 1.5 (light yellow).

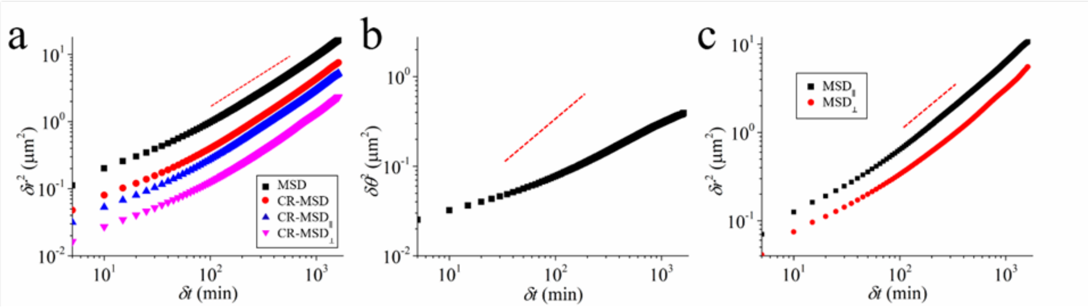

Fig. S3 (a) Cage related TMSD of cells. The black squares represent the TMSD shown in Fig. 2(b) of the main text. The red circles represent the corresponding cage related TMSD. The blue and pink triangles represent the components of the cage related TMSD along and perpendicular to the long axes of cell nuclei, respectively. (b) Rotational mean squared displacement of cells as a function of time delay. (c) The TMSD of cells along (black) and perpendicular (red) to the long axes of cell nuclei. The red dashed lines in (a), (b) and (c) represent a power-law function with an exponent of 1.

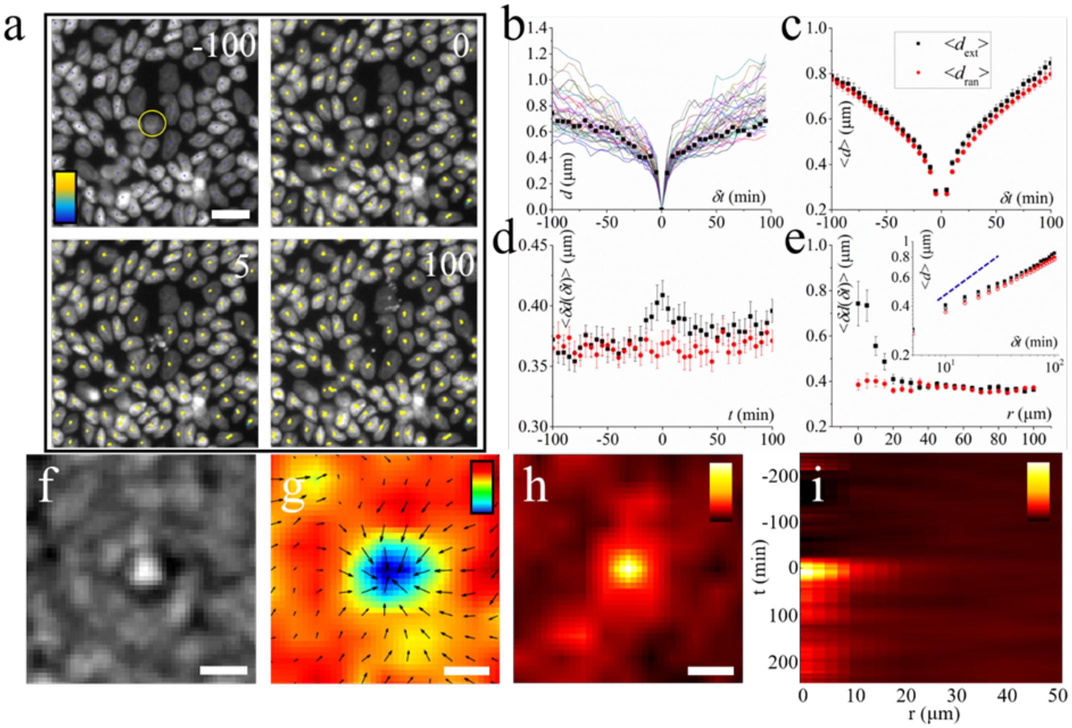

Fig. S4 Dynamics induced by single cell extrusions. (a) Snapshots showing the extrusion of a cell at different moments. The dots represent cell trajectories. The color bar represents time lapse which scales linearly from 0 min (dark blue) to 200 min (light yellow). The extruding cell is marked with a yellow circle. The scale bar is 20 μm. (b) Cell displacement, *d*, averaged over local cells around an extruding cell as a function of time. δt represents time delay before and after extrusion. Solid lines of different colors represent cell displacements of different extrusion events. The black squares represent the cell displacement of the extrusion event in (a). (c) Mean cell displacement, $<d>$, averaged over different extrusion events (black squares, $<d_{ext}>$) as a function of time. The red dots represent the mean cell displacement averaged over different random control regions, $<d_{ran}>$. (d) Mean displacement step, $\langle \delta d(\delta t) \rangle$, of local cells averaged over different extrusion events (black squares) as a function of time before and after extrusion moment. The red dots represent $\langle \delta d(\delta t) \rangle$ of local cells in random square regions. (e) $\langle \delta d(\delta t) \rangle$ at the extrusion moment as a function of distance away from the extrusion event. The black (red) dots represent $\langle \delta d(\delta t) \rangle$ of local cells in extrusion (random) square regions. The inset shows the loglog plot of $d_{ext}(\delta t)$ (black) and $d_{ran}(\delta t)$ (red) in (c) as a function of time. The solid (hollow) symbols represent the mean cell displacements after (before) extrusion. The blue dashed line indicates a power law function with an exponent of 1/2. (f) Microscopic image obtained by averaging the brightfield images of different extrusion events. Scale bar 10 μm (g) Mean velocity field obtained by averaging the PIV (particle image velocimetry) fields of cells around different extrusion events. The velocities are calculated at a time scale of 5 min. Scale bar 20 μm. The color bar represents velocity divergence which scales linearly from -10×10^{-4} (dark blue) to 3×10^{-4} min^{-1} (dark red). (h) 2D spatial pattern of $\langle \delta d(\delta t) \rangle$ at the extrusion moment averaged over different extrusion events. Scale bars 20 μm. (i) Spatiotemporal evolution pattern of $\langle \delta d(\delta t) \rangle$. The color bar scales linearly from 0.3 μm (black) to 0.8 μm (white) in (h) and from 0.3 μm (black) to 1 μm (white) in (i).

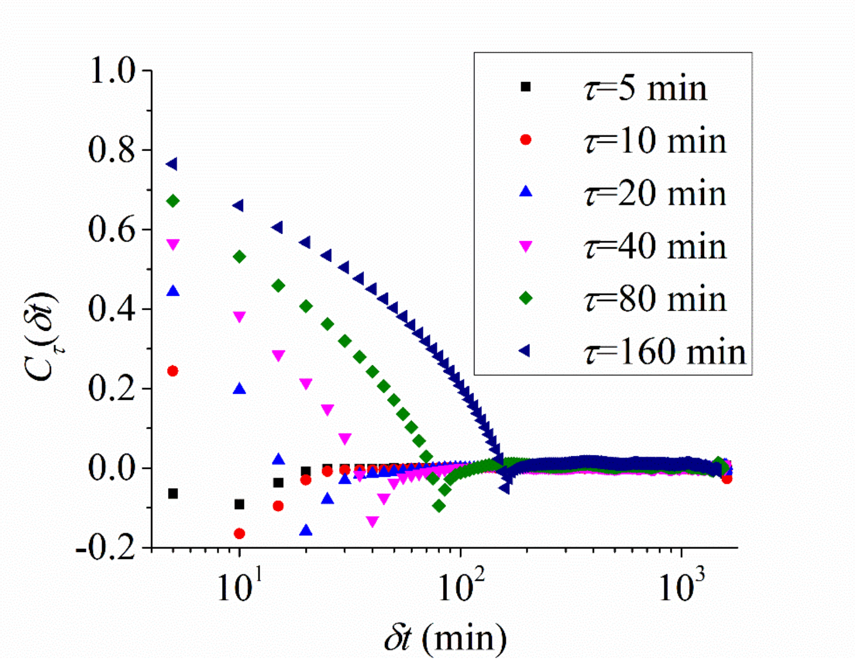

Fig. S5 Time dependence of the autocovariance function of cell motion at different bin times (τ).

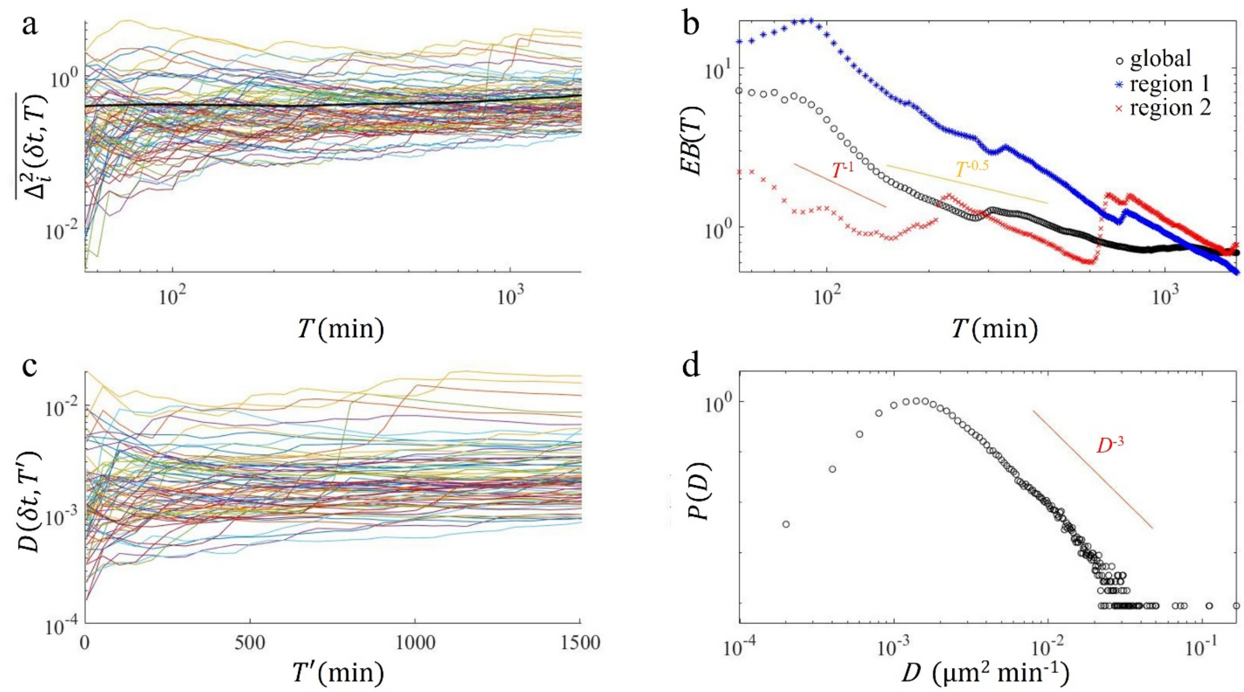

Fig. S6 (a) Individual time averaged MSD curves as a function of time T (only 1/50, randomly selected, cells are shown here). The thick black line represents the time averaged MSD averaged over all cells. (b) The ergodicity breaking parameter (EB) as a function of time. The blue and red data points correspond to cells in two randomly selected local regions (130 μm ×130 μm, including 206 and 189 cells, respectively). The black circles are the data obtained from averaging over the whole sample. (c) Temporal fluctuation of diffusivity D for 77 randomly selected cells. The unit of diffusivity is μm^2/min. (d) The probability density distribution of diffusivity D.

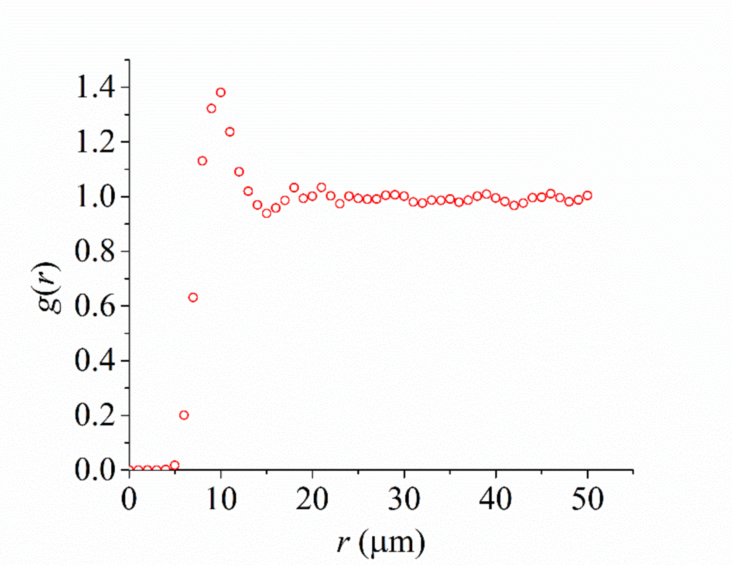

Fig. S7 1D radial distribution function of the epithelial tissue averaged radially in space. The first valley gives a threshold distance $r^{*} = 15$ μm.

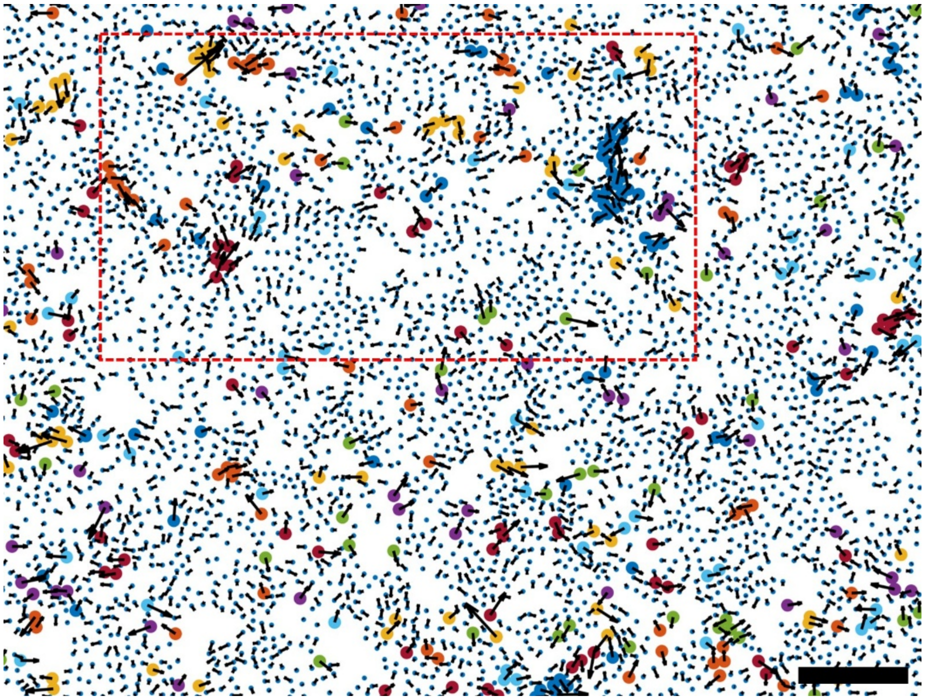

Fig. S8 A snapshot showing the dynamics of cells. The black arrows represent the displacements of cells at t+δt (δt=200 min), which are magnified by 3 times for a better view. The small blue dots represent the positions of cells at t. The large circles of different colors represent different cellular clusters that move quickly (top 10%). The scale bar is 100 μm. The red-dashed square represents the region shown in Fig. 5a.

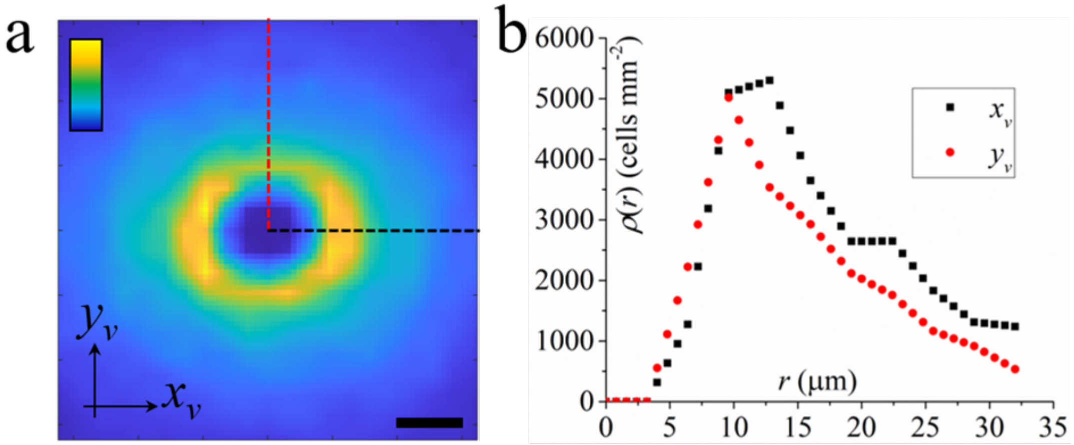

Fig. S9 (a) 2D radial distribution function of cells in large fast cellular clusters in a local coordinate system where the velocity of the reference cell is always aligned along the positive direction of the x_v-axis. The function is not normalized by the mean cell density of the sample, thus the color bar represents cell density which scales linearly from 0 to 6000 (cells per mm^2). Scale bar is 10 μm. (b) The 1D cross profiles of the 2D function along the x_v-axis (black dashed line) and y_v-axis (red dashed line), respectively.

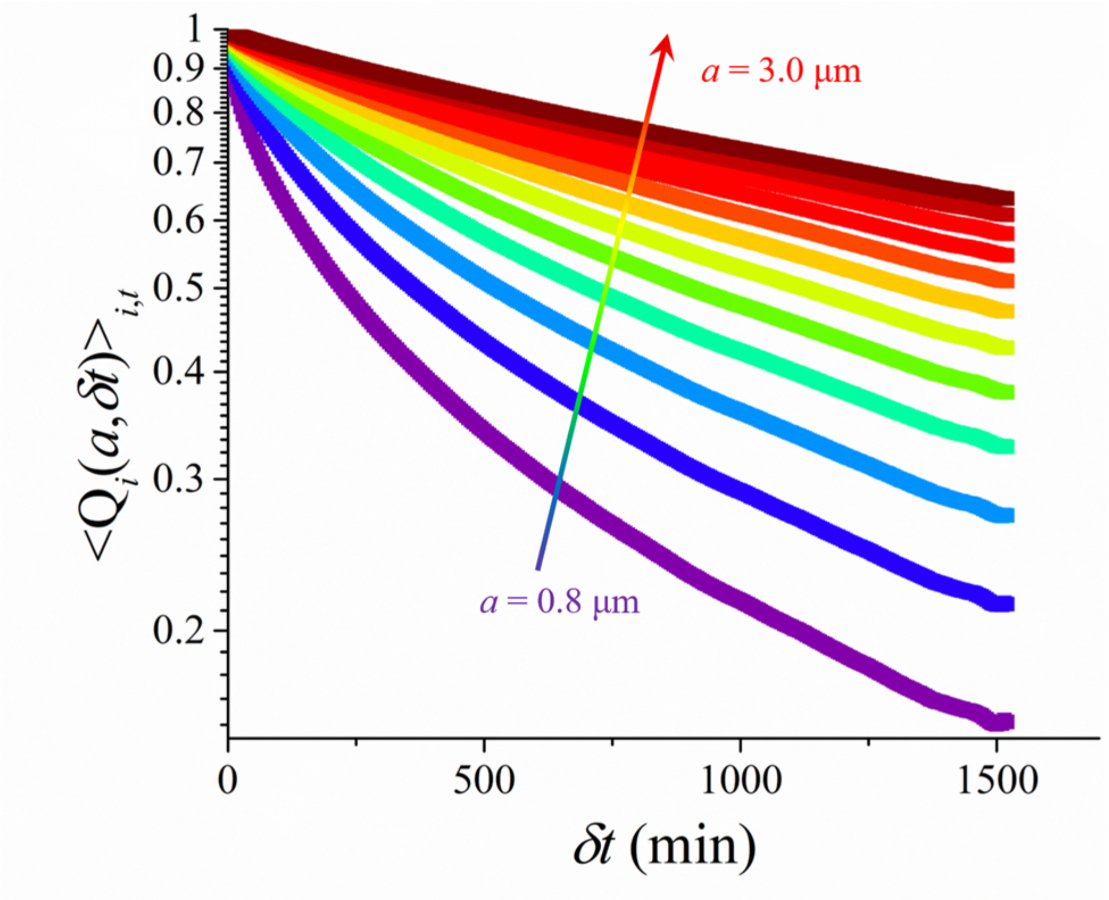

Fig. S10 The temporal dependence of the overlap function. The reference length, a, increases from 0.8 to 3.0 μm in a step of ~0.2 μm as indicated by the arrow.

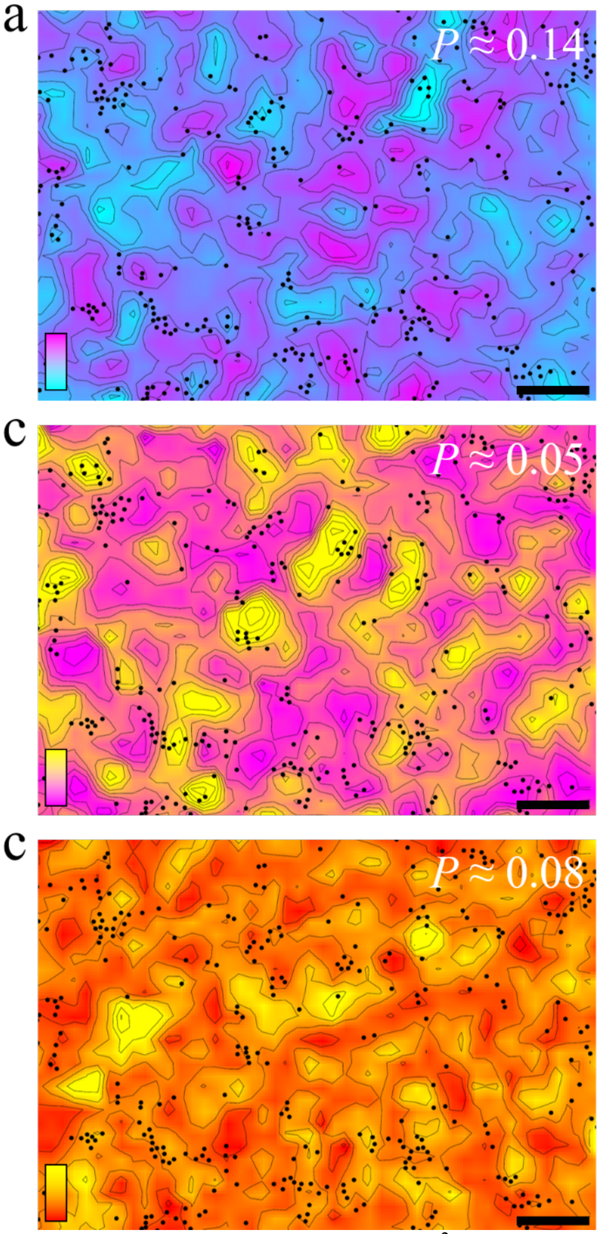

Fig. S11 Contour maps of local density ρ (a), local nematic order O^2 (b), and local hexatic bond orientational order O^6 (c). The black dots represent the cells with the 10% smallest values of self-overlap parameter. The color bars scale linearly from 6×10^{-3} to 13×10^{-3} for ρ, from 0.3 to 0.6 for O^2 and from 0.3 to 0.5 for O^6. Scale bars are 100 µm. P gives the Pearson correlation coefficient and one recognizes that the correlations are weak.

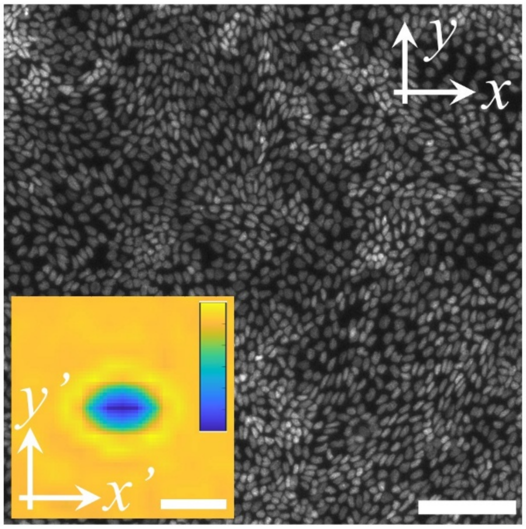

Fig. S12 Replicate of the Fig.1 in the main manuscript showing the spatial arrangement of the cells. Microscopy image of nuclei of MDCK cells at high density. Scale bar is 100 µm. Inset: Two-dimensional pair correlation function of nuclei $g(\mathbf{r})$ in a local coordinate frame with the long-axis of the reference cell nucleus being aligned along the x'-axis. Scale bar is 20 µm. The color bar varies linearly from 0 (dark blue) to 1.2 (light yellow).

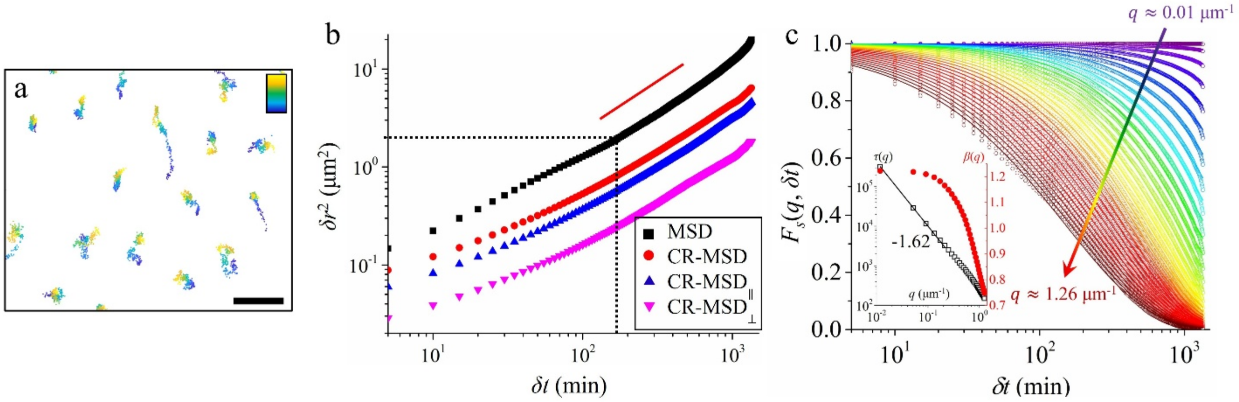

Fig. S13 Replicate of the Fig.2 in the main manuscript showing the microscopic dynamics of cells. (a) Trajectories of cells. Scale bar 10 μm. The color bar scales linearly from dark blue to light yellow, which represents the time lapse from 0 to 1350 min. (b) TMSD of cells (black squares). The red circles represent the corresponding cage related TMSD. The blue and pink triangles represent the components of the cage related TMSD along and perpendicular to the long axes of cell nuclei, respectively. (c) Incoherent intermediate scattering function for different values of the wave vector **q** which increases from ~0.01 to ~1.26 μm^{-1} in a step of ~0.04 μm^{-1} as indicated by the arrow. The solid lines are fits of the form $\exp\left(-\left(\frac{\delta t}{\tau(q)}\right)^{\beta(q)}\right)$. The inset shows $\tau(q)$ and $\beta(q)$ as a function of q.

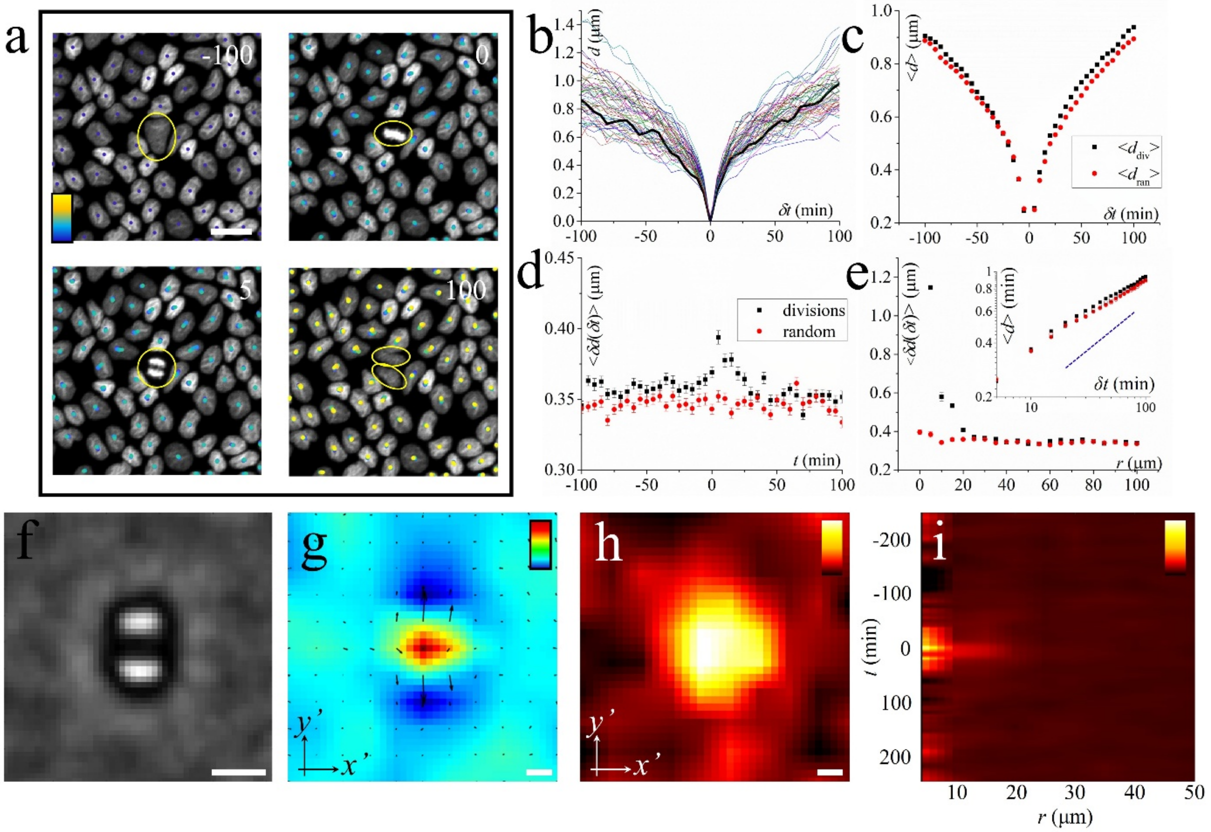

Fig. S14 Replicate of the Fig.3 in the main manuscript showing the local cell dynamics induced by cell divisions. (a) Snapshots showing the division of a cell at different moments. The small dots inside the cells represent cell trajectories. The color bar represents time lapse which scales linearly from 0 min (dark blue) to 100 min (light yellow). The dividing cell is marked with a yellow circle. Scale bar is 20 μm. (b) Cell displacement, *d*, averaged over local cells around a dividing cell (see main text for definition) as a function of time. *δt* represents time delay before and after division. Solid lines of different colors represent cell displacements of different division events. The black thick line represents the cell displacement of the division event in (a). (c) Mean cell displacement, <*d*>, averaged over different division events (black squares, <d_{div}>) as a function of time. The red dots represent the mean cell displacement averaged over different random control regions, <d_{ran}>. (d) Mean displacement step, $\langle \delta d(\delta t)\rangle$, of local cells averaged over different division events (black squares) as a function of time before and after division moment. The red dots represent $\langle \delta d(\delta t)\rangle$ of local cells in random square regions. (e) $\langle \delta d(\delta t)\rangle$ at the division moment as a function of distance away from the division event. The black (red) dots represent $\langle \delta d(\delta t)\rangle$ of local cells in division (random) square regions. The inset shows the log-log plot of $d_{div}(\delta t)$ (black) and $d_{ran}(\delta t)$ (red) in (c) as a function of time. The solid (hollow) symbols represent the mean cell displacements after (before) division. The blue dashed line indicates a power law function with an exponent of 1/2. (f) Microscopic image obtained by averaging the brightfield images of different division events. Each brightfield image is rotated so that the long axis of the condensed chromosome is aligned along the *x*'-axis. Scale bar 10 μm (g) Mean velocity field obtained by averaging the PIV (particle image velocimetry) fields of cells around different division events. The PIV fields are rotated so that the long axis of the condensed chromosome is aligned along the *x*'-axis. The velocities are calculated at a time scale of 5 min. Scale bar 10 μm. The color bar represents the divergence of the velocity field which scales linearly from -2.5×10^{-3} (dark blue) to 4.5×10^{-3} min^{-1} (dark red). (h) 2D spatial pattern of $\langle \delta d(\delta t)\rangle$ at the division moment averaged over different division events. The patterns of $\langle \delta d(\delta t)\rangle$ are rotated so that the long axis of the condensed chromosome is aligned along the *x*'-axis. Scale bar 10 μm. (i) Spatiotemporal evolution pattern of $\langle \delta d(\delta t)\rangle$. The color bars scale linearly from 0.3 μm (black) to 0.6 μm (white) in (h) and from 0.25 μm (black) to 1.2 μm (white) in (i).

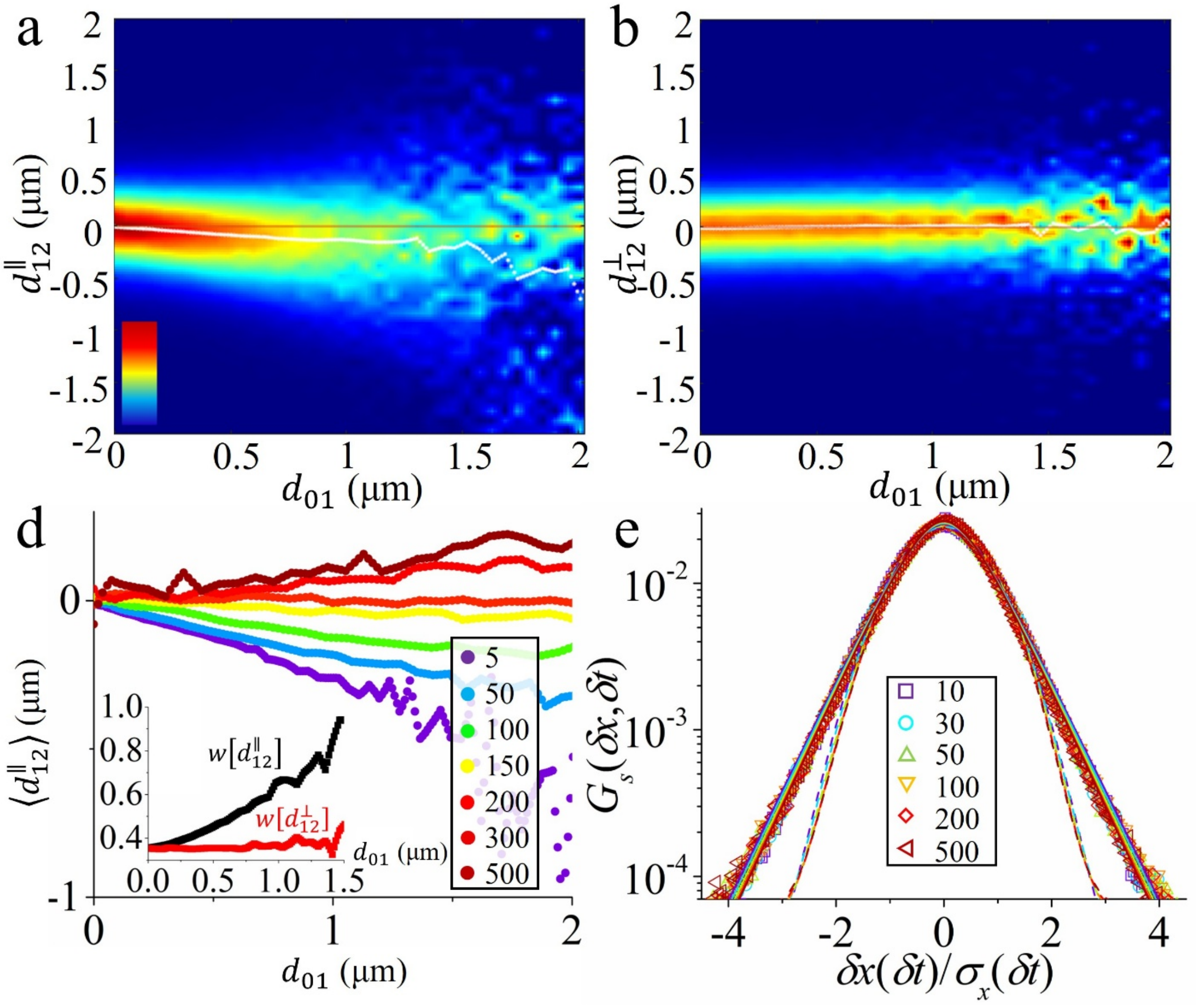

Fig. S15 Replicate of the Fig.4 in the main manuscript showing the creep motion of individual cells. (a) and (b) Conditional probabilities $P\left(d_{12}^{\parallel}|d_{01};\delta t = 10\text{ min}\right)$ and $P(d_{12}^{\perp}|d_{01};\delta t = 10\text{ min})$ (see text for definition). The red solid lines are guides for eyes showing $d_{12}^{\parallel} = 0$ and $d_{12}^{\perp} = 0$. The white lines represent the mean values of $d_{12}^{\parallel}$ and $d_{12}^{\perp}$ as a function of d_{01}. The color bar represents the probability density which scales linearly from 0 (dark blue) to 0.15 μm^{-1} (dark red). (c) The mean values of $d_{12}^{\parallel}$ at different δt as a function of d_{01}. Inset: the FWHM of $P\left(d_{12}^{\parallel}|d_{01};\delta t = 10\text{ min}\right)$ (black) and $P(d_{12}^{\perp}|d_{01};\delta t = 10\text{ min})$ (red) shown in (a) and (b) as a function of d_{01}. (d) Self-part of the Van Hove function for δx (normalized by the square root of the mean squared displacement) at different δt (expressed in minutes). The dashed and solid lines are fits with a Gaussian and a Gumbel law, respectively.

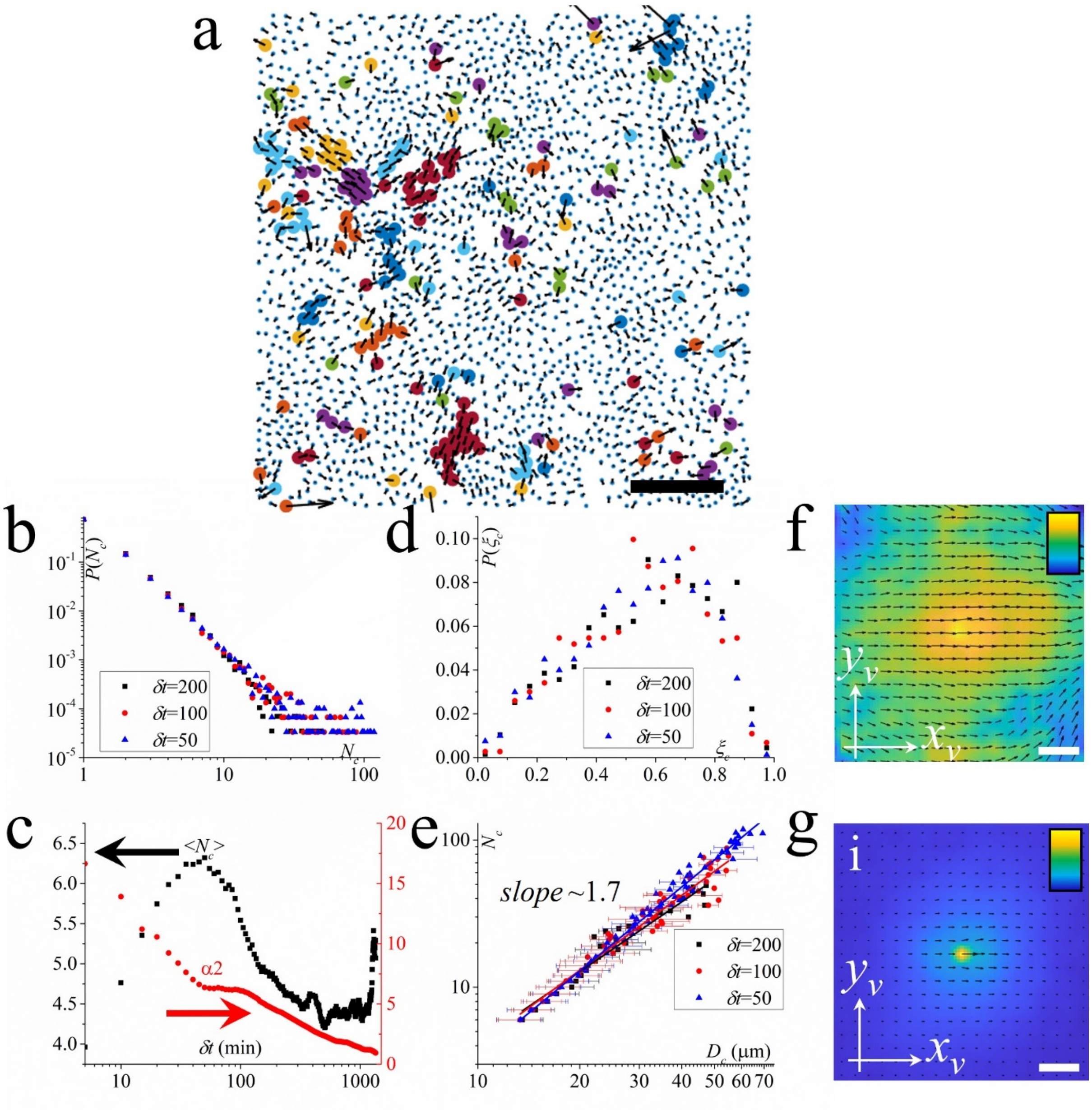

Fig. S16 Replicate of the Fig.5 in the main manuscript showing the dynamics and structure of fast cellular clusters. (a) A snapshot showing the dynamics of cells. The black arrows represent the displacements of cells at t+δt (δt=200 min), which are magnified by 3 times for a better view. The small blue dots represent the positions of cells at t. The large circles of different colors represent different cellular clusters that move quickly (top 10%). The scale bar is 100 μm. (b) Probability density function of cluster size, N_c, for different time δt. (c) Mean size of cellular clusters $<N_c>$ (black) and non-Gaussian parameter α_2 (red) as a function of time step δt. (d) Probability density function of anisotropy of cellular clusters, ξ_c. (e) Double logarithmic plot of cluster size N_c as a function of cluster diameter D_c. The solid black, red and blue lines are power law fits having exponents 1.57, 1.69 and 1.89, respectively. 2D velocity correlation functions of cells in fast clusters (f) and all cells (g) in a local coordinate system where the velocity of the reference cell is always aligned along the positive direction of the x_v-axis. The color bars scale linearly from 0 (dark blue) to 1 (light yellow). Scale bars are 20 μm. The black arrows represent the mean velocity field.

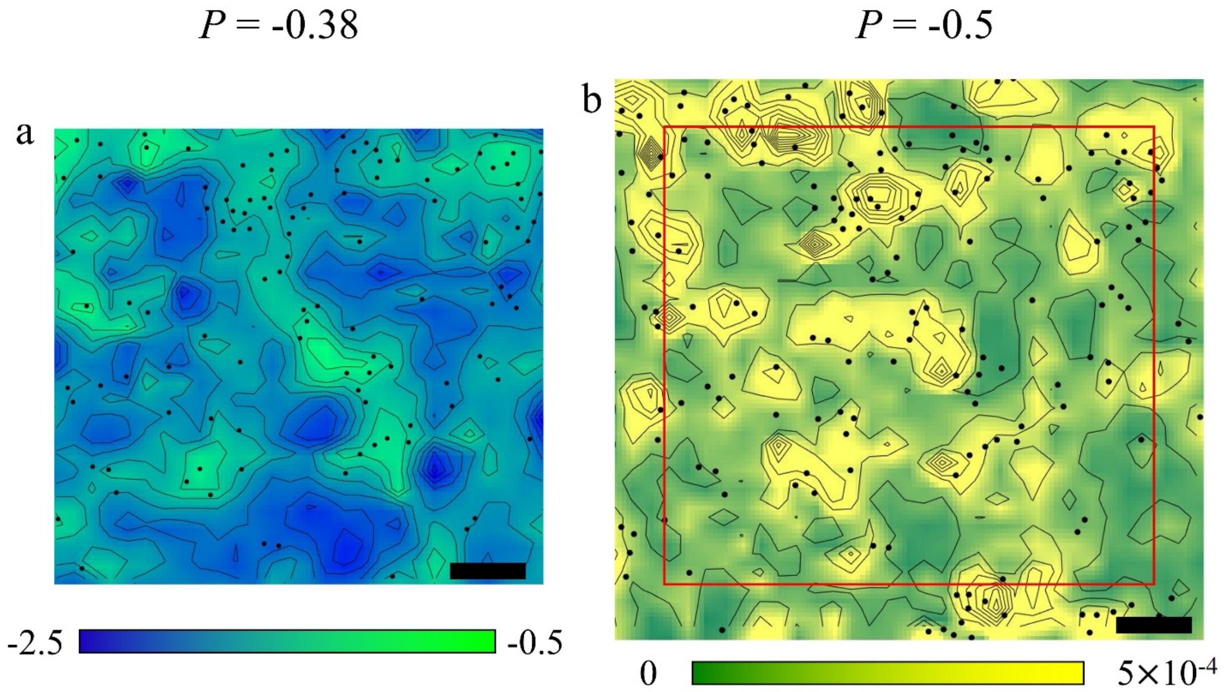

Fig. S17 Replicate of the Fig.6 in the main manuscript showing the spatial correlation between local properties and cell mobility. (a) Contour maps of local structural entropy, *S*, (a) and mean magnitude of low-frequency normal modes, <*M*>, (b). The black dots represent the mobile cells with the 10% smallest values of self-overlap parameter $Q_i(a, \delta t)$. The color bars scale linearly from -2.5 to -0.5 for *S* and from 0 to 5×10^{-4} for <*M*>. Scale bars are 100 μm. The contour map of *S* is only calculated within the red dashed square region of the contour map of <*M*> to exclude the boundary effect. The Pearson correlation coefficients are P = -0.38 and P = -0.5, respectively.

REFERENCES

1. P. Friedl, D. Gilmour, Collective cell migration in morphogenesis, regeneration and cancer. *Nat. Rev. Mol. Cell Biol.* **10**, 445–457 (2009).

2. P.-F. Lenne, V. Trivedi, Sculpting tissues by phase transitions. *Nat. Commun.* **13**, 664 (2022).

3. L. Oswald, S. Grosser, D. M. Smith, J. A. Käs, Jamming transitions in cancer. *J. Phys. D Appl. Phys.* **50**, 483001 (2017).

4. M. Sadati, N. T. Qazvini, R. Krishnan, C. Y. Park, J. J. Fredberg, Collective migration and cell jamming. *Differentiation* **86**, 121–125 (2013).

5. S. Henkes, Y. Fily, M. C. Marchetti, Active jamming: Self-propelled soft particles at high density. *Phys. Rev. E* **84**, 040301 (2011).

6. T. E. Angelini, E. Hannezo, X. Trepat, M. Marquez, J. J. Fredberg, D. A. Weitz, Glass-like dynamics of collective cell migration. *Proc. Natl. Acad. Sci. U.S.A.* **108**, 4714–4719 (2011).

7. J. P. Garrahan, Dynamic heterogeneity comes to life. *Proc. Natl. Acad. Sci.U.S.A.* **108**, 4701–4702 (2011).

8. B. Ladoux, R.-M. Mège, Mechanobiology of collective cell behaviours. *Nat. Rev. Mol. Cell Biol.* **18**, 743–757 (2017).

9. E. Blauth, H. Kubitschke, P. Gottheil, S. Grosser, J. A. Käs, Jamming in embryogenesis and cancer progression. *Front. Phys.* **9**, 666709 (2021).

10. C. Blanch-Mercader, V. Yashunsky, S. Garcia, G. Duclos, L. Giomi, P. Silberzan, Turbulent dynamics of epithelial cell cultures. *Phys. Rev. Lett.* **120**, 208101 (2018).

11. S.-Z. Lin, W.-Y. Zhang, D. Bi, B. Li, X.-Q. Feng, Energetics of mesoscale cell turbulence in two-dimensional monolayers. *Commun. Phys.* **4**, 21 (2021).

12. Y. Shen, J. O'Byrne, A. Schoenit, A. Maitra, R.-M. Mège, R. Voituriez, B. Ladoux, Flocking and giant fluctuations in epithelial active solids. *Proc. Natl. Acad. Sci. U.S.A.* **122**, e2421327122 (2025).

13. S. R. K. Vedula, M. C. Leong, T. L. Lai, P. Hersen, A. J. Kabla, C. T. Lim, B. Ladoux, Emerging modes of collective cell migration induced by geometrical constraints. *Proc. Natl. Acad. Sci. U.S.A.* **109**, 12974–12979 (2012).

14. A. J. Kabla, Collective cell migration: Leadership, invasion and segregation. *J. R. Soc. Interface* **9**, 3268–3278 (2012).

15. R. Alert, X. Trepat, Physical models of collective cell migration. *Annu. Rev. Condens. Matter Phys.* **11**, 77–101 (2020).

16. W. Kang, J. Ferruzzi, C.-P. Spatarelu, Y. L. Han, Y. Sharma, S. A. Koehler, J. A. Mitchel, A. Khan, J. P. Butler, D. Roblyer, A novel jamming phase diagram links tumor invasion to non-equilibrium phase separation. *Iscience* **24**, 103252 (2021).

17. O. Ilina, P. G. Gritsenko, S. Syga, J. Lippoldt, C. A. La Porta, O. Chepizhko, S. Grosser, M. Vullings, G.-J. Bakker, J. Starruß, Cell–cell adhesion and 3D matrix confinement determine jamming transitions in breast cancer invasion. *Nat. Cell Biol.* **22**, 1103–1115 (2020).

18. A. Mongera, P. Rowghanian, H. J. Gustafson, E. Shelton, D. A. Kealhofer, E. K. Carn, F. Serwane, A. A. Lucio, J. Giammona, O. Campàs, A fluid-to-solid jamming transition underlies vertebrate body axis elongation. *Nature* **561**, 401–405 (2018).

19. N. I. Petridou, B. Corominas-Murtra, C.-P. Heisenberg, E. Hannezo, Rigidity percolation uncovers a structural basis for embryonic tissue phase transitions. *Cell* **184**, 1914–1928. e19 (2021).

20. S. Kim, R. Amini, S.-T. Yen, P. Pospíšil, A. Boutillon, I. A. Deniz, O. Campàs, A nuclear jamming transition in vertebrate organogenesis. *Nat. Mater.* **23**, 1592–1599 (2024).

21. M. A. Mendieta-Serrano, Y. Hou, S. Theis, T. E. Hall, S. E. Taylor, B. Verd, R. G. Parton, T. E. Saunders, A structural transition ensures robust formation of skeletal muscle. bioRxiv 677369 [Preprint] (2025); www.biorxiv.org/content/10.1101/2025.09.22.677369v1.

22. O. Chepizhko, M. C. Lionetti, C. Malinverno, C. Giampietro, G. Scita, S. Zapperi, C. A. La Porta, From jamming to collective cell migration through a boundary induced transition. *Soft Matter* **14**, 3774–3782 (2018).

23. S. Garcia, E. Hannezo, J. Elgeti, J.-F. Joanny, P. Silberzan, N. S. Gov, Physics of active jamming during collective cellular motion in a monolayer. *Proc. Natl. Acad. Sci. U.S.A.* **112**, 15314–15319 (2015).

24. D. Bi, X. Yang, M. C. Marchetti, M. L. Manning, Motility-driven glass and jamming transitions in biological tissues. *Phys. Rev. X* **6**, 021011 (2016).

25. E. Hannezo, C.-P. Heisenberg, Rigidity transitions in development and disease. *Trends Cell Biol.* **32**, 433–444 (2022).

26. E.-M. Schoetz, M. Lanio, J. A. Talbot, M. L. Manning, Glassy dynamics in three-dimensional embryonic tissues. *J. R. Soc. Interface* **10**, 20130726 (2013).

27. J.-A. Park, J. H. Kim, D. Bi, J. A. Mitchel, N. T. Qazvini, K. Tantisira, C. Y. Park, M. McGill, S.-H. Kim, B. Gweon, Unjamming and cell shape in the asthmatic airway epithelium. *Nat. Mater.* **14**, 1040–1048 (2015).

28. J. Jiang, Z. Zeng, Z. Pan, B. Shi, Y. Wang, H. Zhang, Collective dynamics of gastric cancer cells in fluid. *Phys. Rev. E* **104**, 064402 (2021).

29. K. Binder, W. Kob, *Glassy Materials and Disordered Solids: An Introduction to their Statistical Mechanics* (World scientific, 2011).

30. D. Bi, J. H. Lopez, J. M. Schwarz, M. L. Manning, Energy barriers and cell migration in densely packed tissues. *Soft Matter* **10**, 1885–1890 (2014).

31. D. Bi, J. Lopez, J. M. Schwarz, M. L. Manning, A density-independent rigidity transition in biological tissues. *Nat. Phys.* **11**, 1074–1079 (2015).

32. Y.-W. Li, L. L. Y. Wei, M. Paoluzzi, M. P. Ciamarra, Softness, anomalous dynamics, and fractal-like energy landscape in model cell tissues. *Phys. Rev. E* **103**, 022607 (2021).

33. S. Sadhukhan, S. K. Nandi, Theory and simulation for equilibrium glassy dynamics in cellular Potts model of confluent biological tissue. *Phys. Rev. E* **103**, 062403 (2021).

34. S. Pandey, S. Kolya, P. Devendran, S. Sadhukhan, T. Das, S. K. Nandi, The structure-dynamics feedback mechanism governs the glassy dynamics in epithelial monolayers. *Soft Matter* **21**, 269–276 (2025).

35. M.-Y. Li, Y.-W. Li, Relaxation dynamics in the self-propelled Voronoi model for epithelial monolayers. *Phys. Rev. Res.* **6**, 033209 (2024).

36. S. Sadhukhan, S. Dey, S. Karmakar, S. K. Nandi, A perspective on active glassy dynamics in biological systems. *Eur. Phys. J. Spec. Top.* **233**, 3193–3224 (2024).

37. M. Czajkowski, D. M. Sussman, M. C. Marchetti, M. L. Manning, Glassy dynamics in models of confluent tissue with mitosis and apoptosis. *Soft Matter* **15**, 9133–9149 (2019).

38. J. Ranft, M. Basan, J. Elgeti, J.-F. Joanny, J. Prost, F. Jülicher, Fluidization of tissues by cell division and apoptosis. *Proc. Natl. Acad. Sci. U.S.A.* **107**, 20863–20868 (2010).

39. D. A. Matoz-Fernandez, K. Martens, R. Sknepnek, J.-L. Barrat, S. Henkes, Cell division and death inhibit glassy behaviour of confluent tissues. *Soft Matter* **13**, 3205–3212 (2017).

40. B. Doliwa, A. Heuer, Cage effect, local anisotropies, and dynamic heterogeneities at the glass transition: A computer study of hard spheres. *Phys. Rev. Lett.* **80**, 4915–4918 (1998).

41. E. R. Weeks, D. Weitz, Properties of cage rearrangements observed near the colloidal glass transition. *Phys. Rev. Lett.* **89**, 095704 (2002).

42. C. A. Angell, K. L. Ngai, G. B. McKenna, P. F. McMillan, S. W. Martin, Relaxation in glassforming liquids and amorphous solids. *J. Appl. Phys.* **88**, 3113–3157 (2000).

43. B. Wang, S. M. Anthony, S. C. Bae, S. Granick, Anomalous yet brownian. *Proc. Natl. Acad. Sci. U.S.A.* **106**, 15160–15164 (2009).

44. B. Wang, J. Kuo, S. C. Bae, S. Granick, When Brownian diffusion is not Gaussian. *Nat. Mater.* **11**, 481–485 (2012).

45. R. Pastore, A. Ciarlo, G. Pesce, F. Greco, A. Sasso, Rapid Fickian yet non-Gaussian diffusion after subdiffusion. *Phys. Rev. Lett.* **126**, 158003 (2021).

46. A. G. Cherstvy, O. Nagel, C. Beta, R. Metzler, Non-Gaussianity, population heterogeneity, and transient superdiffusion in the spreading dynamics of amoeboid cells. *Phys. Chem. Chem. Phys.* **20**, 23034–23054 (2018).

47. A. D. Fernández, P. Charchar, A. G. Cherstvy, R. Metzler, M. W. Finnis, The diffusion of doxorubicin drug molecules in silica nanoslits is non-Gaussian, intermittent and anticorrelated. *Phys. Chem. Chem. Phys.* **22**, 27955–27965 (2020).

48. Z. W. Wu, W. Kob, W.-H. Wang, L. Xu, Stretched and compressed exponentials in the relaxation dynamics of a metallic glass-forming melt. *Nat. Commun.* **9**, 5334 (2018).

49. L. Cipelletti, L. Ramos, Slow dynamics in glassy soft matter. *J. Phys. Condens. Matter* **17**, R253–R285 (2005).

50. L. Ramos, L. Cipelletti, Ultraslow dynamics and stress relaxation in the aging of a soft glassy system. *Phys. Rev. Lett.* **87**, 245503 (2001).

51. P. Ballesta, A. Duri, L. Cipelletti, Unexpected drop of dynamical heterogeneities in colloidal suspensions approaching the jamming transition. *Nat. Phys.* **4**, 550–554 (2008).

52. G. Marty, O. Dauchot, Subdiffusion and cage effect in a sheared granular material. *Phys. Rev. Lett.* **94**, 015701 (2005).

53. M. Weiss, Single-particle tracking data reveal anticorrelated fractional Brownian motion in crowded fluids. *Phys. Rev. E* **88**, 010101 (2013).

54. D. Molina-Garcia, T. Sandev, H. Safdari, G. Pagnini, A. Chechkin, R. Metzler, Crossover from anomalous to normal diffusion: Truncated power-law noise correlations and applications to dynamics in lipid bilayers. *New J. Phys.* **20**, 103027 (2018).

55. B. Kou, Y. Cao, J. Li, C. Xia, Z. Li, H. Dong, A. Zhang, J. Zhang, W. Kob, Y. Wang, Granular materials flow like complex fluids. *Nature* **551**, 360–363 (2017).

56. Y. Yuan, Z. Zeng, Y. Xing, H. Yuan, S. Zhang, W. Kob, Y. Wang, From creep to flow: Granular materials under cyclic shear. *Nat. Commun.* **15**, 3866 (2024).

57. M. D. Ediger, Spatially heterogeneous dynamics in supercooled liquids. *Annu. Rev. Phys. Chem.* **51**, 99–128 (2000).

58. A. Heuer, K. Okun, Heterogeneous and homogeneous dynamics in a simulated polymer melt: Analysis of multi-time correlation functions. *J. Chem. Phys.* **106**, 6176–6186 (1997).

59. W. K. Kegel, A. van Blaaderen, Direct observation of dynamical heterogeneities in colloidal hard-sphere suspensions. *Science* **287**, 290–293 (2000).

60. E. R. Weeks, J. C. Crocker, A. C. Levitt, A. Schofield, D. A. Weitz, Three-dimensional direct imaging of structural relaxation near the colloidal glass transition. *Science* **287**, 627–631 (2000).

61. A. S. Keys, A. R. Abate, S. C. Glotzer, D. J. Durian, Measurement of growing dynamical length scales and prediction of the jamming transition in a granular material. *Nat. Phys.* **3**, 260–264 (2007).

62. O. Dauchot, G. Marty, G. Biroli, Dynamical heterogeneity close to the jamming transition in a sheared granular material. *Phys. Rev. Lett.* **95**, 265701 (2005).

63. D. Stauffer, A. Aharony, *Introduction to Percolation Theory* (Taylor & Francis, 2018).

64. H.-S. Niwa, School size statistics of fish. *J. Theor. Biol.* **195**, 351–361 (1998).

65. E. Bonabeau, L. Dagorn, P. Freon, Scaling in animal group-size distributions. *Proc. Natl. Acad. Sci. U.S.A.* **96**, 4472–4477 (1999).

66. H.-P. Zhang, A. Be'er, E.-L. Florin, H. L. Swinney, Collective motion and density fluctuations in bacterial colonies. *Proc. Natl. Acad. Sci. U.S.A.* **107**, 13626–13630 (2010).

67. I. D. Couzin, J. Krause, Self-organization and collective behavior in vertebrates. *Adv. Stud. Behav.* **32**, 1–75 (2003).

68. R. Großmann, L. S. Bort, T. Moldenhawer, M. Stange, S. S. Panah, R. Metzler, C. Beta, Non-Gaussian displacements in active transport on a carpet of motile cells. *Phys. Rev. Lett.* **132**, 088301 (2024).

69. W. Wang, Y. Liang, A. V. Chechkin, R. Metzler, Non-Gaussian behavior in fractional Laplace motion with drift. *Phys. Rev. E* **111**, 034121 (2025).

70. O. P. Pishnyak, S. V. Shiyanovskii, O. D. Lavrentovich, Inelastic collisions and anisotropic aggregation of particles in a nematic collider driven by backflow. *Phys. Rev. Lett.* **106**, 047801 (2011).

71. T. A. Witten, L. M. Sander, Diffusion-limited aggregation. *Phys. Rev. B* **27**, 5686–5697 (1983).

72. D. He, N. Ekere, L. Cai, Two-dimensional percolation and cluster structure of the random packing of binary disks. *Phys. Rev. E* **65**, 061304 (2002).

73. D. R. Nelson, *Defects And Geometry in Condensed Matter Physics* (Cambridge Univ. Press, 2002).

74. H. Tanaka, T. Kawasaki, H. Shintani, K. Watanabe, Critical-like behaviour of glass-forming liquids. *Nat. Mater.* **9**, 324–331 (2010).

75. P. Patel, M. Sharma, S. M. Bhattacharyya, Dynamic heterogeneity in polydisperse systems: A comparative study of the role of local structural order parameter and particle size. *J. Chem. Phys.* **159**, 044501 (2023).

76. A. Widmer-Cooper, H. Perry, P. Harrowell, D. R. Reichman, Irreversible reorganization in a supercooled liquid originates from localized soft modes. *Nat. Phys.* **4**, 711–715 (2008).

77. A. Widmer-Cooper, H. Perry, P. Harrowell, D. R. Reichman, Localized soft modes and the supercooled liquid's irreversible passage through its configuration space. *J. Chem. Phys.* **131**, 194508 (2009).

78. A. Ghosh, V. Chikkadi, P. Schall, D. Bonn, Connecting structural relaxation with the low frequency modes in a hard-sphere colloidal glass. *Phys. Rev. Lett.* **107**, 188303 (2011).

79. S. Henkes, C. Brito, O. Dauchot, Extracting vibrational modes from fluctuations: A pedagogical discussion. *Soft Matter* **8**, 6092–6109 (2012).

80. D. T. Tambe, C. Corey Hardin, T. E. Angelini, K. Rajendran, C. Y. Park, X. Serra-Picamal, E. H. Zhou, M. H. Zaman, J. P. Butler, D. A. Weitz, Collective cell guidance by cooperative intercellular forces. *Nat. Mater.* **10**, 469–475 (2011).

81. Y. Goswami, G. Shivashankar, S. Sastry, Yielding behaviour of active particles in bulk and in confinement. *Nat. Phys.* **21**, 817–824 (2025).

82. R. Sharma, S. Karmakar, Activity-induced annealing leads to a ductile-to-brittle transition in amorphous solids. *Nat. Phys.* **21**, 253–261 (2025).

83. E. Falck, T. Róg, M. Karttunen, I. Vattulainen, Lateral diffusion in lipid membranes through collective flows. *J. Am. Chem. Soc.* **130**, 44–45 (2008).

84. J.-H. Jeon, H. M.-S. Monne, M. Javanainen, R. Metzler, Anomalous diffusion of phospholipids and cholesterols in a lipid bilayer and its origins. *Phys. Rev. Lett.* **109**, 188103 (2012).

85. J.-H. Jeon, M. Javanainen, H. Martinez-Seara, R. Metzler, I. Vattulainen, Protein crowding in lipid bilayers gives rise to non-Gaussian anomalous lateral diffusion of phospholipids and proteins. *Phys. Rev. X* **6**, 021006 (2016).

86. W. He, H. Song, Y. Su, L. Geng, B. J. Ackerson, H. Peng, P. Tong, Dynamic heterogeneity and non-Gaussian statistics for acetylcholine receptors on live cell membrane. *Nat. Commun.* **7**, 11701 (2016).

87. L. Atia, J. J. Fredberg, N. S. Gov, A. F. Pegoraro, Are cell jamming and unjamming essential in tissue development? *Cells Dev.* **168**, 203727 (2021).

88. S. J. Stehbens, E. Scarpa, M. D. White, Perspectives in collective cell migration–moving forward. *J. Cell Sci.* **137**, jcs261549 (2024).

89. C. Stringer, T. Wang, M. Michaelos, M. Pachitariu, Cellpose: A generalist algorithm for cellular segmentation. *Nat. Methods* **18**, 100–106 (2021).

90. J.-Y. Tinevez, N. Perry, J. Schindelin, G. M. Hoopes, G. D. Reynolds, E. Laplantine, S. Y. Bednarek, S. L. Shorte, K. W. Eliceiri, TrackMate: An open and extensible platform for single-particle tracking. *Methods* **115**, 80–90 (2017).

91. Y. Shen, I. Dierking, Electrically driven formation and dynamics of skyrmionic solitons in chiral nematics. *Phys. Rev. Appl.* **15**, 054023 (2021).

92. P.-G. De Gennes, J. Prost, *The Physics of Liquid Crystals* (Oxford Univ. Press, ed. 2, 1993), vol. 83.

93. K. J. Naidoo, J. Schnitker, Melting of two-dimensional colloidal crystals: A simulation study of the Yukawa system. *J. Chem. Phys.* **100**, 3114–3121 (1994).

94. A. Ghosh, V. K. Chikkadi, P. Schall, J. Kurchan, D. Bonn, Density of states of colloidal glasses. *Phys. Rev. Lett.* **104**, 248305 (2010).

95. E. Yamamoto, T. Akimoto, A. Mitsutake, R. Metzler, Universal relation between instantaneous diffusivity and radius of gyration of proteins in aqueous solution. *Phys. Rev. Lett.* **126**, 128101 (2021).
